# Integrin-KCNB1 potassium channel complexes regulate neocortical neuronal development and are implicated in epilepsy

**DOI:** 10.1038/s41418-022-01072-2

**Published:** 2022-10-07

**Authors:** Alessandro Bortolami, Wei Yu, Elena Forzisi, Koray Ercan, Ritik Kadakia, Madhuvika Murugan, Denise Fedele, Irving Estevez, Detlev Boison, Mladen-Roko Rasin, Federico Sesti

**Affiliations:** 1 Department of Neuroscience and Cell Biology, Robert Wood Johnson Medical School, Rutgers University, Piscataway, NJ USA; 2 Department of Neurosurgery, Robert Wood Johnson Medical School, Rutgers University, Piscataway, NJ USA; 3 Department of Cell Biology and Neuroscience, School of Arts and Sciences, Rutgers University, Piscataway, NJ USA

**Keywords:** Experimental models of disease, Integrins

## Abstract

Potassium (K^+^) channels are robustly expressed during prenatal brain development, including in progenitor cells and migrating neurons, but their function is poorly understood. Here, we investigate the role of voltage-gated K^+^ channel KCNB1 (Kv2.1) in neocortical development. Neuronal migration of glutamatergic neurons was impaired in the neocortices of KCNB1 null mice. Migratory defects persisted into the adult brains, along with disrupted morphology and synaptic connectivity. Mice developed seizure phenotype, anxiety, and compulsive behavior. To determine whether defective KCNB1 can give rise to developmental channelopathy, we constructed Knock In (KI) mice, harboring the gene variant *Kcnb1*^*R312H*^ (R312H mice) found in children with developmental and epileptic encephalopathies (DEEs). The R312H mice exhibited a similar phenotype to the null mice. Wild type (WT) and R312H KCNB1 channels made complexes with integrins α5β5 (Integrin_K^+^ channel_Complexes, IKCs), whose biochemical signaling was impaired in R312H brains. Treatment with Angiotensin II in vitro, an agonist of Focal Adhesion kinase, a key component of IKC signaling machinery, corrected the neuronal abnormalities. Thus, a genetic mutation in a K^+^ channel induces severe neuromorphological abnormalities through non-conducting mechanisms, that can be rescued by pharmacological intervention. This underscores a previously unknown role of IKCs as key players in neuronal development, and implicate developmental channelopathies in the etiology of DEEs.

## Introduction

The voltage-gated K^+^ channel KCNB1, forms macromolecular complexes with integrins, named Integrin_K^+^ channel_Complexes (IKCs) [1–3]. Evidence suggests that IKCs regulate fundamental cellular functions, such as migration, proliferation, survival and death through non-conducting (non-ionic) mechanisms [1]. The crucial role of IKCs is further underscored by the fact that mutations in the *KCNB1* gene are found in children affected by developmental and epileptic encephalopathies (DEEs), neurological conditions characterized by severe developmental delays, that often co-exist with seizures and abundant epileptiform abnormalities [4, 5]. While the mechanisms underlying DEEs are currently under investigation, accumulating evidence points out to abnormal neuronal development and resulting synaptic connectivity disturbances, as one of the underlying causes [6, 7]. It is not coincidental that in vitro IKCs formed with certain KCNB1 DEE-variants, impair cellular functions such as migration and neuritogenesis, through non-ionic mechanisms [2]. Given the established importance of integrins in determining neocortical developmental processes, these findings support an argument that IKCs may be implicated in prenatal brain development and consequently, that developmental channelopathies may contribute to the etiology of DEEs [2, 8–12].

To elucidate the role of IKCs in the mechanisms governing the development of the brain and more broadly, their impact on neurological disease, we generated a *knock in* (KI) mouse model of DEEs harboring the *Kcnb1*^*R312H*^ gene variant, and a null (NULL) mouse bearing a null allele encoding a 337 amino acids truncated KCNB1 protein. The NM_004975.2:c.935G>A mutation, which causes an arginine to histidine replacement in the voltage sensor of the channel (R312H), was initially identified in two children affected by severe developmental delays and severe epilepsy that was highly refractory to anti-epileptic drugs [7].

Here, we report that IKCs are essential for the migration of glutamatergic neurons during prenatal brain development that they control through non-ionic functions. Accordingly, IKC formed with R312H KCNB1 subunits impair neuronal migration causing significant non-structural abnormalities in the adult brain, and are associated with epilepsy and behavioral deficit.

## Methods and materials

### Reagents and resources

**Table:** 

Reagent or resource	Source	Identifier
Antibodies		
p44/42 MAP kinase (phosphorylated Erk1/2)	Cell Signaling Technology	Cat# 9101, RRID:AB_331646
p44/42 MAPK (Erk1/2) (137F5) Rabbit mAb	Cell Signaling Technology	Cat# 4695, RRID:AB_390779
Phospho-MEK1/2 (Ser217/221) (41G9) Rabbit mAb	Cell Signaling Technology	Cat# 9154, RRID:AB_2138017
MEK1/2 Antibody	Cell Signaling Technology	Cat# 9122, RRID:AB_823567
FAK Antibody	Cell Signaling Technology	Cat# 3285, RRID:AB_2269034
Phospho-FAK (Tyr576/577) Antibody	Cell Signaling Technology	Cat# 3281, RRID:AB_331079
Vinculin (E1E9V) XP®	Cell Signaling Technology	Cat# 13901, RRID:AB_2728768
Talin-1 (C45F1) Rabbit mAb	Cell Signaling Technology	Cat# 4021, RRID:AB_2204018
Phospho-Talin (Ser425) Antibody	Cell Signaling Technology	Cat# 5426, RRID:AB_10695406
Paxillin Antibody	Cell Signaling Technology	Cat# 2542, RRID:AB_10693603
Phospho-Paxillin (Tyr118) Antibody	Cell Signaling Technology	Cat# 2541, RRID:AB_2174466
Integrin beta-5 (D24A5) Rabbit mAb	Cell Signaling Technology	Cat# 3629, RRID:AB_2249358
Integrin alpha-5 Antibody	Cell Signaling Technology	Cat# 4705, RRID:AB_2233962
Ctip2 alias Bcl-11B (D6F1) XP® Rabbit mAb	Cell Signaling Technology	Cat# 12120, RRID:AB_2797823
Phospho-Synapsin-1 (Ser605) (D4B9I) Rabbit mAb	Cell Signaling Technology	Cat# 88246, RRID:AB_2800119
PSD-95 (D74D3) XP Rabbit mAb	Cell Signaling Technology	Cat# 3409, RRID:AB_1264242
ILK1 (4G9) Rabbit mAb	Cell Signaling Technology	\Cat# 3856, RRID:AB_2233861
MAP2 Antibody	Cell Signaling Technology	Cat# 4542, RRID:AB_10693782
Src Antibody	Cell Signaling Technology	Cat# 2108, RRID:AB_331137
Anti-c-Raf Antibody	Cell Signaling Technology	Cat# 9422, RRID:AB_390808
Anti-Actin Antibody, clone C4	Millipore	Cat# MAB1501, RRID:AB_2223041
Anti-Synapsin I Antibody	Millipore	Cat# AB1543, RRID:AB_2200400
Anti-Potassium Channel Kv2.1	Millipore	Cat# AB5186-50UL, RRID:AB_91734
Synapsin-1 Monoclonal Antibody (7H10G6)	Thermo Fisher Scientific	Cat# MA5-31919, RRID:AB_2787542
Goat anti-Mouse IgG (H + L) Cross-Adsorbed Secondary Antibody, Alexa Fluor™ 488	Thermo Fisher Scientific Millipore	Cat# A-11001, RRID:AB_2534069
Goat anti-Rabbit IgG (H + L) Highly Cross-Adsorbed Secondary Antibody, Alexa Fluor™ 594	Thermo Fisher Scientific Millipore	Cat# A-11037, RRID:AB_2534095
Goat anti-Chicken IgY (H + L) Cross-Adsorbed Secondary Antibody, Alexa Fluor™ Plus 64	Thermo Fisher Scientific Millipore	Cat# A32933, RRID:AB_2762845
Alpha Parvin/Actopaxin antibody	Proteintech	Cat# 11202-1-AP, RRID:AB_2236617
SATB2 antibody [SATBA4B10]	Abcam	Cat# ab51502, RRID:AB_882455
Vinculin (phospho Y821) antibody	Abcam	Cat# ab61071, RRID:AB_946347
Chemicals		
Akt inhibitor class IV	Santa Cruz Biotechnology	Cat# sc-203809
Angiotensin II Human	Millipore	Cat# A9525-5MG
PAF C-16	ChemCruz	Cat# sc-201009A
Cilenginide/Cyclo (-RGDfK)	APExBIO	Cat# A8164
PND-1186	APExBIO	Cat# A3730
Commercial assays		
FD Rapid GolgiStain Kit	FD NeuroTechnologies.INC	Cat# PK401A
MTT Assay Kit	Abcam	Cat# ab211091
Ras Pull-down Activation Assay Biochem Kit	Cytoskeleton	Cat# BK008
Protein A/G PLUS-Agarose beads	Santa Cruz Biotechnology	Cat# sc-2003
Experimental models: Cell lines		
Hamster: CHO cells	Sesti Lab	N/A
Mouse: N2a	Sesti Lab	N/A
Organisms/strains		
Mouse: C57BL6/J background	The Jackson Laboratory	Strain #:000664 RRID:IMSR_JAX:000664
Mouse: KI *Kcnb1*^*R312H*^	Genome Editing Core Facility at Rutgers	R312H mouse (provisional)
Mouse: null mouse	Genome Editing Core Facility at Rutgers	NULL mouse (provisional)
Recombinant DNA		
Src dominant negative mutant K295R	Dr. Frank Suprynowicz	Src K295R
WT KCNB1-HA	Sesti Lab	N/A
R312H KCNB1-HA	Sesti Lab	N/A
pDEST27-Vinculin	Addgene	20144
pEGFP_WT-talin1 (1-2541)	Addgene	166112
Mek1	Addgene	40774
Mek2	Addgene	40776
Flag-Paxillin	Addgene	15212
pLL3.7m-psRaf1	Addgene	89364
RAF1 gRNA (BRDN0001148013)	Addgene	76706
pLenti-puro/RAF1-S259A	Addgene	131727
pcDNA3-T4-ERK1	Addgene	14440
pcDNA3-HA-ERK2 WT	Addgene	8974
HA-Akt DN (K179M)	Addgene	16243
pcDNA3-mAkt-ER	Addgene	39530
Software and algorithms		
Neuroanatomy Fiji ImageJ	Tiago Ferreira	https://imagej.net/update-sites/neuroanatomy/
EthoVision XT	N/A	https://www.noldus.com/ethovision-xt
Colocalization-Threshold Fiji ImageJ	Tony Collins	https://imagej.net/plugins/colocalization-threshold
Prism	GraphPad by Dotmatics	https://www.graphpad.com/scientific-software/prism/
Image J	NIH	https://imagej.net/software/fiji/

### Construction of R312H and NULL KI mice

The Knock In mouse harboring *Kcnb1*^*R312H*^ (NM_004975.2:c.935G>A:p.R312H) in the C57BL6/J background, named R312H mouse, was constructed by the Genome Editing Core Facility at Rutgers using CRISPR technology. In generating the KIs we made null alleles and frameshifts that were not repaired correctly. The NULL mice used in this study bears a null allele which encodes a 337 amino acids truncated KCNB1 protein. All strains have been backcrossed two generations.

### In vivo animal studies

Mice of either sexes at developmental stages: E13, P0, P7, and 3 month old was used. Littermates of either sexes were randomly assigned (by flipping of a coin) to experimental groups. Animals were housed in an AAALAC approved vivarium under the care of a veterinarian. The animals were housed in large cages, with a 12-h light-dark cycle, and fed ad libitum. We adhere and thus followed the guiding principles of animal care as approved by the American Physiological Society, the Guide for the Care of Laboratory Animals and our institution’s Animal Care and Use Committee (IACUC). All experiments with animals performed in this study were IACUC approved.

### EGG recording and behavioral protocols

EGG recordings and behavioral tests were performed with prior knowledge of the genotype.

#### Open field

We followed a standard protocol as described by Seibenhener and Wooten [13]. Briefly, testing was performed in dim light, in a room equipped with a plexiglass arena (50 cm × 50 cm × 40 cm) whose walls were covered by tissue paper to prevent the mouse from seeing outside, and a video-tracking system (EthoVision XT; Noldus Information Technology, Leesburg, VA). The mouse was allowed to acclimate to the procedure room for 30 min. Then the mouse was placed in the middle of the arena and the test administrator leaved the room. The mouse was kept in the arena for a single 10 min period during which movement was recorded.

#### Self grooming

Mice were recorded for 6 h with time-locked Lorex cameras and DVR. The overall time spent grooming was divided by the duration of the recording and expressed as the fraction of time spent grooming per minute.

#### EEG recordings and seizure analysis

Eight-nine week old animals heterozygous and homozygous in *Kcnb1*^*R312H*^ and NULL mice were equipped with a bipolar electrode surgically implanted into the hippocampus (AP: −1.94; ML:−1.75, DV: −1.6, relative to bregma), a surface cortical monopolar screw electrode and cerebellum reference screw electrode while under 1.5% isoflurane anesthesia. The mice were allowed to recover for 1 week before the EEG recording. Mice were tethered for the EEG recording for a period of 24 h. Electrical brain activity was amplified (Dual Bio Amp amplifier) and digitized by Powerlab 16/35 data acquisition device using Labchart application interface (ADInstuments, Dunedin, New Zealand). For the seizure analysis, the EEG recordings were systematically reviewed, and the seizures were manually scored and defined as sustained rhythmic synchronous discharges in the EEG, clearly distinguished from background EEG and interictal activity as described previously [14]. Brief bursts of spikes and periodic spikes were not considered part of a seizure.

### Biochemistry

Western blotting was performed with prior knowledge of the genotype. Immunofluorescence and Golgi staining and Sholl analysis, were performed without prior knowledge of the genotype.

#### Crude brain lysates

Half sagittal brains were homogenized with a plastic tissue grinder in lysis buffer (0.32 M sucrose, 5 mM Tris-Cl pH 6.8, 0.5 mM EDTA, and protease inhibitor cocktail (Sigma-Aldrich). The brains samples were further homogenized by being passed 5–6 times with a syringe and/or sonicated for 1–3 min and then centrifuged for 10 min at 14000 rpm at 4 °C. The supernatant was collected and stored at −80 °C for further analyses.

#### Immunoprecipitations

To minimize unspecific binding, lysates were pre-incubated with Protein A/G PLUS-Agarose beads (Santa Cruz Biotechnology) for 20 min at room temperature. The beads were then collected by centrifugation 5000 RPM for 3 min and discharged. The lysates were incubated with the primary antibody overnight at 4 °C. Pre-washed beads were then added to the lysate and incubated at 4 °C for 1 h. Beads were blocked with BSA for 1 h. The beads were washed 4 times with lysis buffer and (0.32 M sucrose, 5 mM Tris-Cl pH 6.8, 0.5 mM EDTA) and collected by centrifugation at 5000 RPM for 3 min. The pellet was resuspended in 3X Sample Buffer and boiled for 5 min at 95–100 °C. The samples were centrifuged 10,000 RPM for 2 min and the supernatant was resolved in an SDS-PAGE for Western blot analysis.

#### Western blotting

For Western blot analysis, protein content, typically 40–100 μg, was quantified by the Bradford colorimetric assay (Sigma-Aldrich). Samples were dissolved in 5X sample buffer (Sodium dodecyl sulfate (SDS) 10%; Bromophenol blue 0.02%; glycerol 30%; Tris-HCL 0.5 M) and 2–5% Beta-mercaptoethanol (Sigma-Aldrich); heated at 95–100 °C for 5 min and then centrifuged at 10,000 RPM for 1 min. The proteins were resolved in 10–15% SDS-PAGE and transferred into a nitrocellulose membrane. Membranes were washed one time in Tris Buffer Saline (TBS) and then blocked in 5% solution of nonfat dry milk in Tris Buffered Saline with Tween® 20 (TBST) for 1 h. Then, membranes were stained with the primary antibody diluted at 1:1000 at 4 °C overnight. After washing 30 min with TBST, membrane were incubated with secondary antibody (Goat Anti-Rabbit IgG Antibody, (H + L) HRP conjugate or Anti-Mouse IgG antibody, dilution 1:4000) for 1 h at room temperature. Membranes were washed for 30 min in TBST and then exposed with SuperSignal™ West Pico PLUS Chemiluminescent Substrate (ThermoFischer).

#### Membrane stripping

Co-IP experiments with integrin-α5, integrin-β5, Vinculin, Talin-1, and ILK were performed on separate membranes and replicated using membrane stripping. Nitrocellulose membranes, after the first chemiluminescence exposure, were washed with Mild Stripping Buffer (1.5% glycine, 0.1% SDS, 1% Tween 20, pH to 2.2) for 20 min, with PBS for 20 min and TBST for 10 min at room temperature. The membranes were blocked with 5% solution of nonfat dry milk in TBST for 1 h and then stained with the primary antibody as described in the *Western Blotting* section.

### Immunofluorescence

Immunofluorescence experiments were performed without prior knowledge of the genotype.

#### Neonatal brains

The detailed biochemical procedures were previously described [15, 16]. Briefly 5-chloro-2-deoxyuridine (CldU) solution at a concentration of 5 ml/kg of mouse weight in sterile PBS, from a 10 mg/mL stock, was prepared freshly and was intraperitoneally injected into pregnant dams at E17. The P7 anesthetized pups (ketamine/xylazine) were washed with 0.9% filtered NaCl and then perfused with 4% paraformaldehyde solutions. Brains were removed, post-fixed in 4% paraformaldehyde overnight at 4 °C, and cryoprotected in 30% sucrose. Brains were embedded in agarose, and slices were prepared throughout the cortex and the hippocampus in a 1:6 series (70 μm thick) using a vibratome (Leica VT1000S), so that the same set of tissue samples could be used for expression of different makers. Samples were incubated in 1.0 M HCl for 30 min under gentle shaking followed by incubation in 2.0 M HCl for 10 min under gentle shaking and washed 4 times with PBS for 5 min each time under vigorous shaking (antigen retrieval). Slices were incubated in blocking/permeabilization buffer (donkey blocking serum pH = 7.43 + Triton-X 0.4%). Then, free-floating slices were processed for labeling with primary antibodies (according to manufacture recommendations). After 24–48 h incubation at 4 °C in primary antibodies, sections were washed three times with PBS for 10 min each time, followed by application of the appropriate secondary conjugated antibodies. After incubation for 1 h at room temperature, sections were washed three times with PBS.

#### Adult brains

The detailed biochemical procedures were previously described [17]. Briefly anesthetized mice (ketamine/xylazine) were washed with 0.9% filtered NaCl and then perfused with 4% paraformaldehyde solutions. Brains were removed, post-fixed in 4% paraformaldehyde overnight at 4 °C, and cryoprotected in 30% sucrose. Brain slices were cut 20 µm thick throughout the cortex and the hippocampus in a 1:20 series so that the same set of tissue samples could be used for expression of different makers. After permeabilization procedures (7 min in 0.1% Triton-X in PBS) when required, mounted sections were processed for double labeling with primary antibodies. After 24–48 h incubation at 4 °C in primary antibodies, sections were washed three times with PBS for 10 min each time and incubated with the appropriate secondary conjugated antibody. After 1 h at room temperature, the sections were washed three times with PBS.

#### Primary neurons

Cells grown in six wells plates were fixed using a 50% methanol, 50% acetone solution for 20 min on dry ice. Cells were washed 3 times with PBS for 10 min each time, and blocked for 1 h at room temperature with 3% BSA in PBS. After 24–48 h incubation at 4 °C in primary antibodies, the cells were washed three times with PBS, followed by application of the appropriate secondary conjugated antibodies. After 1 h at room temperature, the cells were washed three times with PBS. All slides were mounted in VECTASHIELD Antifade Mounting Medium with DAPI mounting buffer (Vector Laboratories, Burlingame, CA) and stored at 4 °C. Staining was visualized with a Zeiss Axiophot microscope or with an Olympus FV1000MPE (Orangeburg, NY) multi-photon microscope or Nikon Eclipse Ti2 series (Tokyo, Japan) confocal microscope, all equipped with dedicated software.

#### Golgi staining

Golgi staining was performed using the FD rapid GolgiStain kit (FD Neurotechnologies, Columbia, MD) according to manufacturer’s instructions. Brains were embedded in Tissue Freezing Medium, sectioned at a 150 µm thickness, mounted on gelatin precoated slides and photographed with a Zeiss Axio Imager M1 at 100×. Sholl analysis of cropped neurons was performed using ImageJ/Fiji software.

### Cell cultures

#### Chinese hamster ovary cells

Chinese hamster ovary (CHO-K1, simply CHO) cells were maintained at 37 °C in a 95% air/5% CO_2_ humidified incubator. They were typically seeded at a density of 0.5 million cells/mL using a hemocytometer, and grown in Dulbecco Modified Eagle’s medium (DMEM) as described [2]. The cells were transfected using Lipofectamine 2000 according to manufacturer’s instructions. Twenty μl of reagent and 4 μg plasmid DNA were used for each transfection.

#### Mouse neuroblastoma cells

Undifferentiated mouse neuroblastoma N2a cells cells were cultured in DMEM supplemented with 10% fetal bovine serum and 1% sodium pyruvate at 37 °C in a 95% air/5% CO_2_ humidified incubator. They were typically seeded at a density of 0.5 million cells/mL. After reaching ∼60% confluence the cells were transfected with Lipofectamine, incubated in serum-free DMEM for 48 h and then analyzed.

#### Primary cortical neurons

The detailed procedure was previously described. Experiments involving primary neurons were performed without prior knowledge of the genotype [17]. Briefly, cortices were obtained from time-mated embryonic day 13 (E13) embryos (pure cultures) or from P0 pups (co-cultures). Cortical tissue from individual embryos/pups was mechanically triturated and neuron isolated and plated in 12 mm poly-d-lysine-precoated petri dishes (GG-12-1.5-PDL, Neuvitro, Camas, WA) at ~250,000 cells/dish (1.5 ml medium/dish). Cultures were maintained in Plating medium at 37 °C in a 95% air/5% CO2 humidified incubator for 24 h. After 24 h the medium was removed and replaced with Neurobasal medium at 37 °C in a 95% air/5% CO2 humidified incubator. Ear samples from individual embryos or pups were processed for genotyping.

### In vitro assays

In vitro experiments were performed without prior knowledge of the genotype.

#### Treatment with Ang II and PAF C-16 agonists

Ang II and PAF C-1 were freshly diluted from ethanol stocks (Ang II: 4.7 mM and PAF C-16: 20 mM) and  added to the medium at the indicated concentrations. In the wound healing assay, Ang II or PAF were added immediately after the scratch and maintained until the end of the experiment. In the N2a assay, PAF or Ang II were added immediately after transfection and maintained until the end of the experiment. In primary neuron cultures, Ang II was added to the medium on DIV2 and maintained until the end of the experiment.

#### Proliferation

CHO cells were seeded into 96-well tissue culture plates in equal numbers. Twenty-four hours after seeding, the cells (∼250,000 cells/well), were transfected with various plasmids using Lipofectamine. One and two days post-transfection (dpt1 and dpt2), the numbers of viable cells were assessed using the microtiter-plate colorimetric MTT assay. Briefly, cells were incubated in serum-free media and MTT reagent for 3 h at 37 °C. Then, MTT solvent was added, the cells were incubated for additional 15 min and then measurements taken using a Tecan Infinite M200pro (Männedorf, Switzerland) microplate reader (590 nm absorbance).

#### Wound healing assay

CHO cells were seeded into 6-well tissue culture plates in equal numbers. Twenty-four hours after seeding, the CHO cells (∼75% confluency), were transfected with Lipofectamine as already described. The monolayer was perpendicularly scratched twice with a 200 μl (yellow) pipette tip. The well was washed with DMEM medium to remove the detached cells and the scratches were photographed with a Zeiss Axiovert 200 M (Oberkochen, Germany) microscope at ×10 magnification. After 24 h the scratches were re-photographed. The gap covered in 24 h was measured using ImageJ 1.52a software (National Institutes of Health, Bethesda, MA). Gap distance was calculated as:

1 $$Gap \,=\, 100\left( {1 \,-\, \frac{{d_{24}}}{{d_0}}} \right)$$

where d_0_ and d_24_ are the gap widths at the time of the scratch and 24 h later. Two technical replicates per time point, per experiment.

#### Neurite outgrowth

Forty-eight hours after transfection cells were photographed with a Zeiss Axiovert 200 M microscope equipped with GFP lamp. The neurite (the longer one in multineuritic cells) and the area of the soma of GFP-fluorescent cells was measured using ImageJ 1.52a software.

#### Electrophysiology

Data were recorded with an Axopatch 200B (Molecular Devices, San Jose, CA), a PC (Dell, Round rock, TX) and Clampex software (Molecular Devices, San Jose, CA) and filtered at f_c_ = 1 kHz and sampled at 2.5 kHz. An Ag-AgCl electrode was connected to the bath solution using a KCl-agar bridge. Bath solution was (in mM): 4 KCl, 100 NaCl, 10 Hepes (pH = 7.5 with NaOH), 1.8 CaCl_2_ and 1.0 MgCl_2_. Pipette solution: 100 KCl, 10 Hepes (pH = 7.5 with KOH), 1.0 MgCl_2_, 1.0 CaCl_2_, 10 EGTA (pH = 7.5 with KOH). Pipettes (∼5 MΩ) were obtained by pulling borosilicate glass with a Sutter P-97 puller (Sutter Instruments, Novato, CA). Whole-cell currents were evoked by 1 s voltage sweeps from an holding potential of −80 mV to +80 mV in 20 mV increments and leak subtraction was performed digitally using Clampfit software (Molecular Devices). Macroscopic conductance (G) curves were calculated as:

2 $$G \,=\, \frac{I}{{V \,-\, V_{rev.}}}$$

where I is the macroscopic current at steady-state (at the end of the voltage pulse) and V_rev._ is the reversal potential, obtained by linear fitting the current–voltage relationships around the *I* = 0 region. Offset potentials, including series resistance were estimated to be ≤5 mV and were not compensated for when generating current–voltage relationships. G/G_Max_ curves were fitted to the Boltzmann function:

3 $$\frac{G}{{G_{Max}}} \,=\, \frac{1}{{1 \,+\, EXP\left[ {\left( {V_{1/2} \,-\, V} \right)/V_S} \right]}}$$

where *V* is the membrane voltage, *V*_*1/2*_ is the value of the voltage at which Eq. 3 is equal to 0.5 and *V*_*S*_ is the slope coefficient (in mV).

### Statistical analysis

Quantitative data are presented as mean ± standard error of the mean (SEM). The estimated sample size, N, needed to detect a meaningful difference between groups was calculated using power analysis with alpha = 0.05 and power = 0.8 [18]. Normality and log-normality tests (D′ Agostino and Pierson) were calculated for normal distribution. The level of significance, assumed at the 95% confidence limit or greater (*P* < 0.05), was calculated using one-way ANOVA with a Tukey’s post hoc test or two-way ANOVA, with a Dunnett’s post hoc test or a two-sample Kolmogorov–Smirnov, that were computed by Prism software.

## Results

### R312H mice exhibit compulsive behaviors

**Fig. 1:**
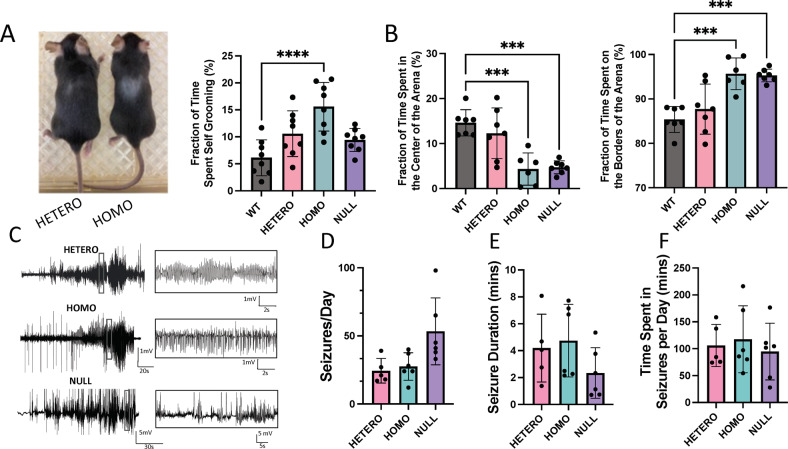
R312H mice exhibit anxious behavior and severe seizure phenotype. **A** Representative pictures of a R312H heterozygote and homozygote mice showing areas of excoriated skin caused by excessive grooming, and percentage of time spent grooming per minute, per genotype (*N* = 8 animals/genotype). Mice were recorded with time-locked Lorex cameras and DVR. **B** Percentage of time spent in the center or on the sides of the arena. Mice were kept in the arena for 10 mins and were recorded using a video-tracking system. WT, HOMO, and NULL: 7 mice/group; HETERO: 6 mice. **C** Representative traces showing spontaneous electrographic seizures recorded from cortical electrodes from mice of the indicated genotypes. **D** Bar graph denotes average number of seizures per day in R312H heterozygotes (*N* = 5), R312H homozygotes (*N* = 6) and NULL (*N* = 6) mice. **E** Mean duration of seizure in seconds in R312H heterozygotes (*N* = 5), R312H homozygotes (*N* = 6) and NULL (*N* = 6) mice. **F** Mean time spent in seizures in a day in minutes in R312H heterozygotes (*N* = 5), R312H homozygotes (*N* = 6) and NULL (*N* = 6) mice. ***P < 0.001 and ****P < 0.0001 (one-way ANOVA, Tukey’s post hoc).

All KI animals were grossly normal in appearance. However, R312H homozygotes (HOMO) did not mate, and were obtained by breeding of heterozygous (HETERO) pairs. The analysis of time-locked video recordings revealed excessive self grooming in the KI population, which often caused skin excoriations (Fig. 1A), and may reflect defects at the level of the neocortex [19]. Compulsive behaviors are associated with anxiety in mice. When tested in the open field maze, R312H homozygotes and NULL spent ∼10-fold more time on the sides than in the center of the arena compared to WT (Fig. 1B) [13]. In contrast, the heterozygotes behaved similarly to control animals, even though the former showed a trend toward more anxiety.

### R312H KI mice are affected by frequent seizures

KI animals exhibited spontaneous convulsive and non-convulsive seizures. Behavioral seizures were both tonic and myoclonic (Supplementary V1). To investigate the epileptic phenotype of the KI animals in more detail, we analyzed EEG recordings in 2–3-month-old mice of either sexes. Both heterozygotes and homozygotes displayed spontaneous electrographic seizures (Fig. 1C). There was no significant difference in seizure frequency or duration across KI mice, so that the seizure burden was comparable in all genotypes (Fig. 1D–F). Thus, a single *Kcnb1*^*R312H*^ allele is sufficient to induce the full seizure phenotype.

### R312H protein is downregulated in the KI brains

**Fig. 2:**
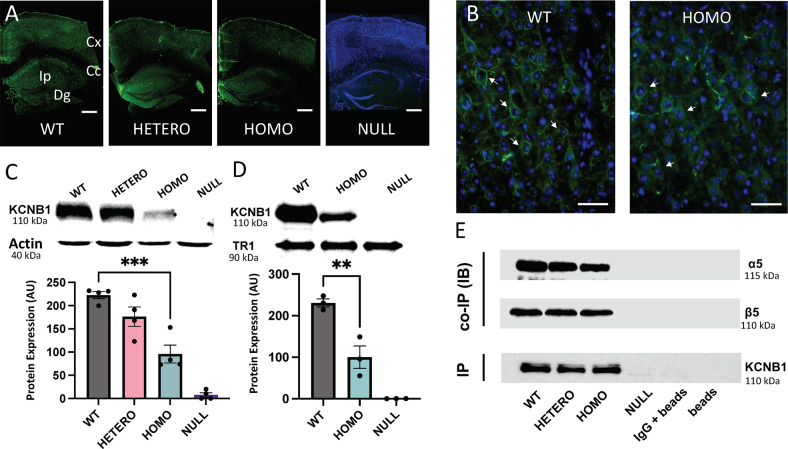
KCNB1 protein is downregulated in the brain. **A** Representative images of brain sections of the indicated genotypes stained with KCNB1 antibody (green). For clarity, DAPI staining, in blue color, is shown only in NULL. Scale bar 800 μm. **B** Magnifications from WT or R312H homozygous cortices demonstrating KCNB1 immunoreactivity in individual neurons. Scale bar 50 μm. **C** Representative Western blots of total KCNB1 or actin (control) for the indicated genotypes and densitometric quantification. *N* = 3 brains/genotype. *P* < 0.001 for comparisons between WT and NULL (not indicated). **D** Representative western blots of surface KCNB1 protein for the indicated genotypes and densitometric quantification. Putative taste receptor 1 (TR1) was used as positive control. *N* = 3 brains/genotype. *P* < 0.001 for comparisons between WT and NULL, (not indicated). **E** Representative co-IPs (IB) of KCNB1 (IP) with integrin-α5 and integrin-β5 from the brains of the indicated genotypes. Controls, mouse IgG and empty beads. Samples were loaded to have approximately the same amount of KCNB1 protein per genotype. The same membrane used to visualize integrin-α5 was stripped and re-blotted with integrin-β5 antibody. **P < 0.01 and ***P < 0.001 (one-way ANOVA, Tukey’s post hoc).

To assess the impact of *Kcnb1*^*R312H*^ on the mouse brain, we characterized the expression of the KCNB1 protein. The neurons reactive to KCNB1 antibody (KCNB1+), were decreased in brain slices of heterozygotes and to a larger extent R312H homozygotes compared to control, and were not detected in NULL mice, as expected (Fig. 2A, B. Bright-field images, Fig. S1A). Accordingly, KCNB1 protein was decreased in Western blots of crude KI brain lysates compared to control, and was absent in NULL mice (Fig. 2C. Uncropped gels in Supplementary Information). The downregulation of R312H protein could be due to defective trafficking to the plasma membrane. However, eye inspection revealed normal expression/distribution of the channel on the membrane of immunoreactive neurons of *Kcnb1*^*R312H*^ genotype (Fig. 2B, arrows). This impression was corroborated by biochemical assessment of surface expression (Fig. 2D), ruling out major trafficking defects (the ratio of R312H/WT protein, total, and at the surface, is ∼0.4 for both).

### R312H channels assemble with integrins

WT KCNB1 channels form macromolecular complexes with integrins in the mouse and human brain [20]. Therefore, we determined whether R312H mutant channels retained the ability to form IKCs. In three experiments, R312H co-immunoprecipitated with integrin-α5 and integrin-β5 (Fig. 2E). We refer to complexes containing R312H subunits as IKC_R312H_. Complexes formed with WT channels are referred to as IKC_WT_ or simply IKCs.

### R312H neuronal development is disrupted in vitro

The low levels of R312H protein may be due to decreased cell viability. Accordingly, immunoreactivity to neuronal marker microtubule-associated protein 2 (Map2), was reduced in the cortices of KI animals, suggesting disrupted neuronal maturation, density or organization—with large areas possibly depleted of pyramidal cells (Fig. S2) [21]. The sick cells may be eliminated during prenatal development, when neocortical progenitors differentiate into Map2+ glutamatergic neurons (KCNB1 is embryonically expressed, Fig. S3, [22]), or they may slowly degenerate and die, or both. To distinguish between these possibilities, we examined pure cultures harvested from the neocortices of embryonic day 13 (E13) embryos (when neocortical wall contains neuronal progenitors), and co-cultures of neurons and glia cells, when all neocortical neurons are already born [23–25]. At day in vitro 3 (DIV3), the number of cells co-stained for KCNB1 and Map2 was comparable in all pure cultures, irrespective of the genotype, as expected (Fig. S4A, C) [26]. In contrast, when compared to WT in co-cultures, the number of cells co-stained for KCNB1 and Map2 was decreased by roughly 20% and 40% in R312H heterozygote and homozygote cells, respectively, (Fig. S4B, D). Notably, the decrease of KCNB1+ cells in co-cultures, matched the decrease of total and surface KCNB1 protein. Neuronal mortality from DIV3 to DIV14 was ∼10–15%, irrespective of the genotype or culture type. At DIV14, in all cultures, KCNB1+ neurons had assumed typical pyramidal cell shape, and likewise in vivo neurons, the R312H variant was normally present and formed clusters, at the surface (Fig. S4E, F; 3-D reconstructions of individual neurons, Supplementary V2). Taken together, these data reveal disrupted neuronal development or loss of neurons expressing KCNB1 in KI animals taking place between E13 and P0, a stage in which the neocortex is formed—suggesting that corticogenesis might be impaired in the KI brains.

### Neuronal migration is hindered in the neocortices of R312H mice

**Fig. 3:**
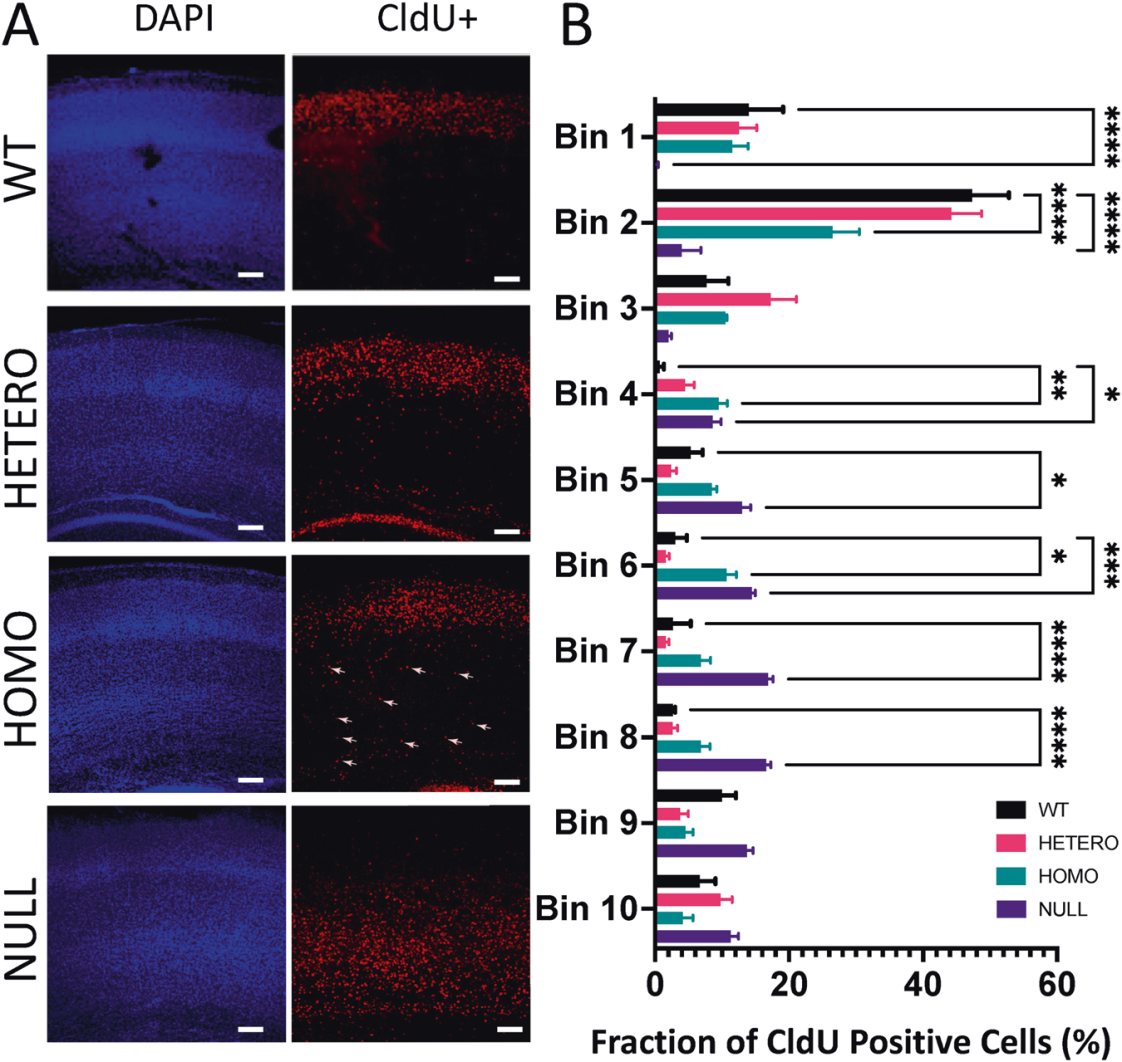
Neuronal migration is impaired in R312H neocortices. **A** Representative confocal images of neocortex at P7 stained for DAPI (blue) and CldU (red). CldU was injected at E17 when upper layer neurons are born. Representative examples of cells stuck in migration are indicated by arrows. Scale bar 200 μm. **B** Neocortex was divided into 10 equal bins and relative distribution of CldU positive cells per bin was graphed. WT: 4 brains; HETERO: 4 brains; HOMO: 4 brains and NULL: 3 brains. *P < 0.05; **P < 0.01; ***P < 0.001 and ****P < 0.0001 (two-way ANOVA, Dunnett’s post hoc).

To begin revealing the role of IKCs in neurodevelopment, we injected thymidine analog CldU at E17, to label progenitors of glutamatergic intracortically projecting neurons and their progenies destined for upper layers (UL). Remarkably, at P7 we found significant numbers of E17 CldU+ neuronal progenies in the deep layers (DL) instead of UL, in heterozygotes and to a larger extent R312H homozygotes (Fig. 3. Bright-field images, Fig. S1B). This migratory defect was further exacerbated in NULL animals, where the majority of E17 born neurons remained in the DL. These data suggest that IKCs are required for neuronal migration to UL in developing neocortices.

### R312H mice exhibit neuromorphological malformations

**Fig. 4:**
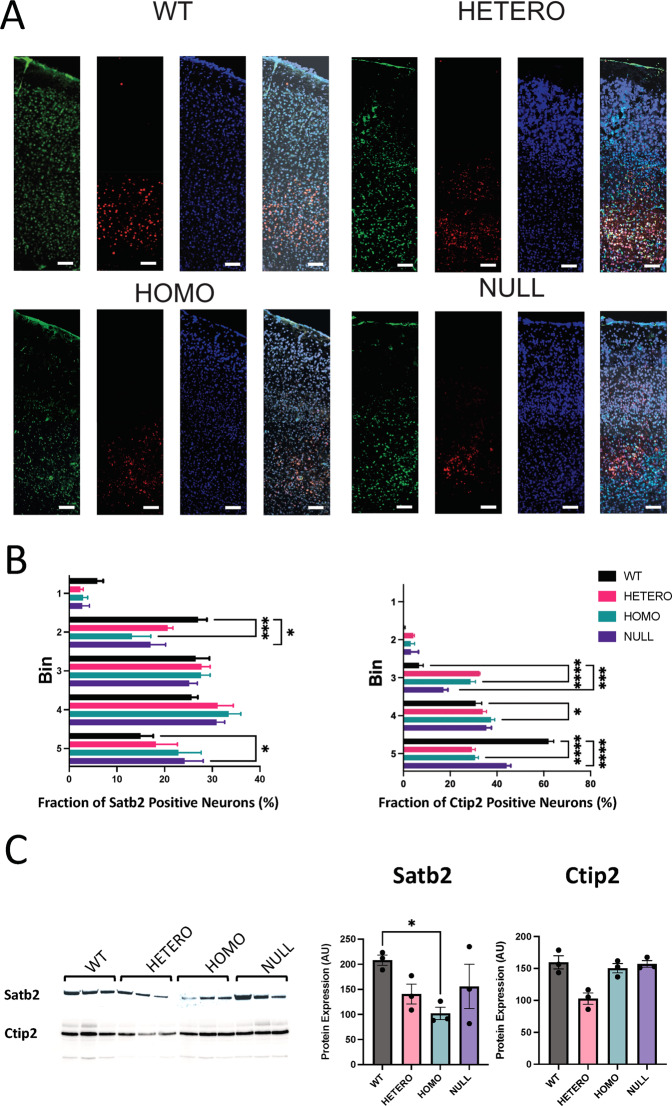
Adult R312H brains exhibit severe neuroabnormalities. **A** Representative images of Satb2 (green), Ctip2 (red) and DAPI (blue) staining and of Satb2 and Ctip2 and DAPI overlaps in central cortex of 3-month-old mice of the indicated genotypes. Scale bar 500 μm. **B** Relative distribution of Satb2, or Ctip2 positive cells per bin. *N* = 6 brains/genotype. Two technical replicates/brain. **C** Representative western blot of Satb2 protein and of Ctip2 in the indicated 3 brains/genotype, and densitometric quantification. Loading controls: Bradford assay. *P < 0.05, ***P < 0.001, and ****P < 0.0001 (two-way ANOVA, Dunnett’s post hoc).

Deficits in neuronal migration are a primary cause of brain malformations, and can be associated with seizures [27]. Therefore, we sought to determine the potential impact of the migratory defects on different subpopulations of neocortical glutamatergic neurons, long-term. Coronal sections of 3-month-old mice were immunostained with Satb2, a transcription factor expressed in intracortically projecting neurons, including UL, regulating their development, and with Ctip2, a transcription factor expressed in subcortically projecting neurons in DL, regulating their development [28, 29]. The cortices of WT mice, consistently exhibited immunoreactivity to Satb2 glutamatergic neurons between layers 2 and 5 (Fig. 4A, B). Ctip2+ neurons were detected only in DL, where they overlapped with Satb2+ cells, as expected (Fig. 4A, B). Conversely, the number of Satb2+ neurons in homozygous R312H, and NULL cortices, was significantly decreased in the ULs (corresponding to bins 1–3 in Fig. 4B) and increased in DLs (bins 4–5), while Ctip2+ neurons were also present in the mid layers. Western blot analysis revealed significant decreases in the levels of Satb2 protein in the brains of homozygotes, whereas the levels of Ctip2 did not significantly vary across genotypes (Fig. 4C).

### Synaptic functionality is impaired in homozygous R312H KI mice

**Fig. 5:**
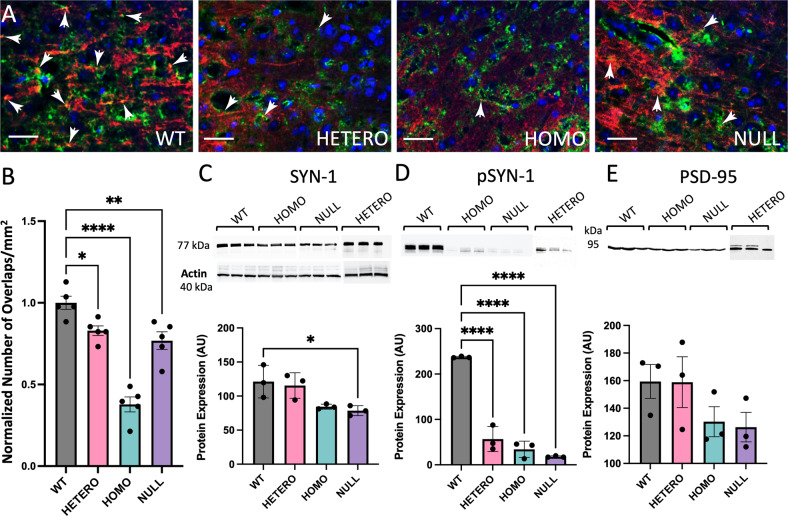
Synaptic connectivity is hindered in R312H cortices. **A** Representative images of SYN-1 (green), PSD-95 (red), and DAPI (blue) co-staining in the cortices of the indicated genotypes. Functional synapses were identified as SYN-1 and PSD-95 signals co-localizations (arrows). Scale bar 25 μm. **B** Quantification of functional synapses, given as the number of SYN-1 and PSD-95 co-localizations. Data are normalized to WT. Sections were analyzed with ImageJ/Fiji software. *N* = 5 brains/genotype. Two technical replicates/brain. **C** Western blots and densitometric quantification of SYN-1 protein in crude brain lysates of the indicated genotypes. *N* = 3 brains/genotype. **D** Western blots and densitometric quantification of SYN-1 protein phosphorylated at S605 (pSYN-1) in crude brain lysates of the indicated genotypes. *N* = 3 brains/genotype. **E** Western blots and densitometric quantification of PSD-95 protein in crude brain lysates of the indicated genotypes. *N* = 3 brains/genotype. The westerns in **C**–**E** were carried out using the same bran lysates. Loading controls were Bradford assay and actin (**C**). *P < 0.05, **P < 0.01, and ****P < *0.0001* (one-way ANOVA, Tukey’s post hoc).

The emergence of functional neocortical connectivity depends on neuronal development driving synaptic formation, and may be affected by neuron viability [30]. Accordingly, immunoreactivity to Synapsin-1 (SYN-1), a pre-synaptic marker was comparable to control in heterozygous sections, but was significantly decreased in the cortices and the hippocampi of homozygous and NULL animals (Fig. S5. See also the immunoblots in Fig. 5C) [31]. Representative examples of cortical sections co-stained with SYN-1 and PSD-95 (a post-synaptic marker), are illustrated in Fig. 5A [32]. We found lower numbers of SYN-1/PSD-95 co-stainings in the KI cortices compared to WT, suggesting a decrease in establishing functional synapses (Fig. 5B). Furthermore, the amounts of SYN-1 protein, total and phosphorylated at Ser605 (pSYN-1, a proxy for functional SYN-1 in vivo [33]), were significantly lower in KI and NULL brains compared to control (Fig. 5C, D). PSD-95 showed a trend toward decreased protein levels in homozygotes and NULL animals (Fig. 5E). Overall, these findings implicate IKCs in neocortical synapses and circuits formation.

### Golgi staining underscores morphology abnormalities in R312H neurons

**Fig. 6:**
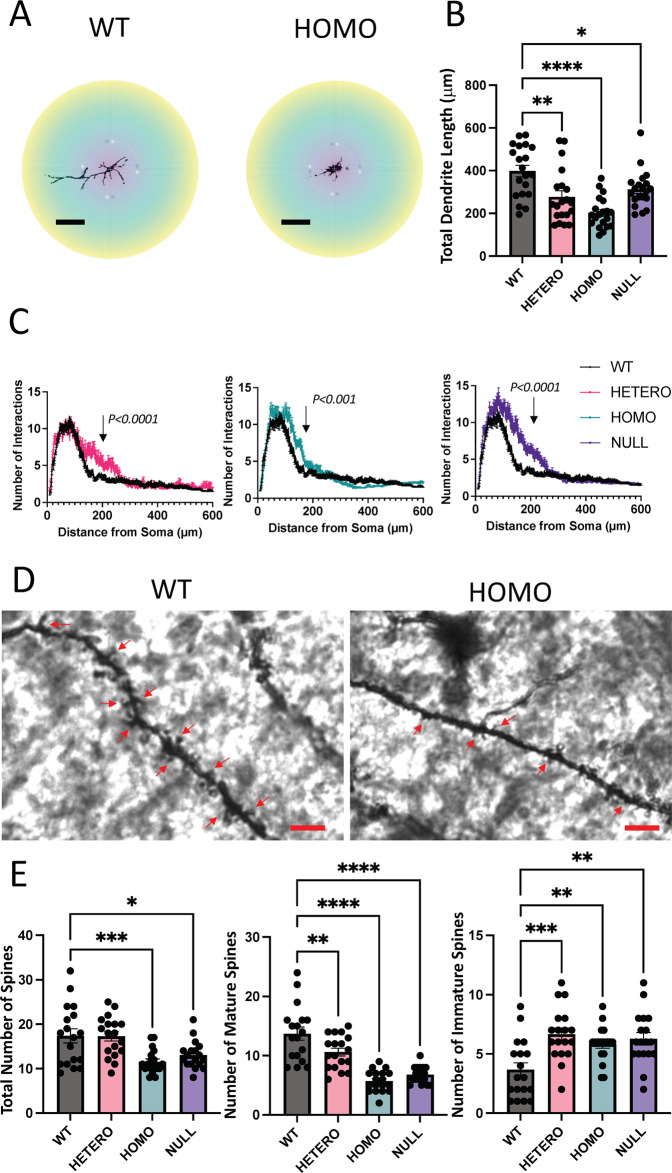
R312H pyramidal cells display morphological defects. **A** Representative images of a WT and a homozygous R312H pyramidal neuron used for Sholl analysis. Individual neurons were cropped and analyzed using ImageJ/Fiji software. Scale bar 50 μm. **B** Total dendrite length. *N* = 20 cells/genotype taken from 3 brains/genotype. **C** Mean Sholl intersection profiles of the neurons of the various genotypes. Each mean SIP was obtained by averaging 20 individual SIPs from 3 brains/genotype. *P* < 0.0001 for WT vs. heterozygous R312H; *P* < 0.001 for WT vs. homozygous R312H and *P* < 0.0001 for WT vs. NULL (Kolmogorov–Smirnov test). **D** Representative images of dendritic spines in a WT and R312H homozygous neuron. Scale bar 10 μm. **E** Number of total, mature and immature spines for the indicated genotypes. For a single neuron, the number of spines was counted over a single dendrite for a continuous length of ∼100 μm. *N* = 20 cells/genotype taken from 3 brains/genotype. *P < 0.05, **P < 0.01, ***P < 0.001, and ****P < 0.0001 (one-way ANOVA, Tukey’s post hoc).

Disrupted neuronal migration and synaptogenesis are likely to be associated with altered morphology of neocortical neurons. To test this, we performed silver Golgi staining of neocortices (Fig. S6). We observed less neurons Golgi impregnated in UL, along with disrupted columnar organization of apical dendrites in homozygotes, NULL and to a lesser extent, heterozygotes. There was accumulation of Golgi impregnated cells in Layer I. Finally, significant numbers of neurons in DL (e.g., layer 5) had apical dendrite disoriented or reversed. Individual KI neurons exhibited shorter apical dendrite and excessive arborizations around the soma (Figs. 6A, S6). To characterize the morphology of KI pyramidal cells, we performed Sholl analysis [34]. The Total Dendrite Length (TDL) was decreased in all KI genotypes (Fig. 6B). The Sholl intersection profiles (SIPs) exhibited typical shape corresponding to the pyramidal cell—with the number of interactions peaking at a shorter distance from the soma (Fig. 6C). However, the SIPs of the KI neurons were broader compared to control, reflecting increased dendritic complexity and abnormal arborization arising from the soma [35]. Furthermore, the dendrites of homozygous neurons had significantly less dendritic spines than WT (Fig. 6D, E), consistent with the lower number of mature synapses evidenced by the SYN-1/PSD-95 co-immunostainings (Fig. 5). Homozygote dendritic spines had immature morphological characteristics—they were thinner compared to control spines, and did not exhibit the typical mushroom-like shape (Fig. 6D). The total number of spines was comparable between heterozygotes and WT, but the former were more immature (Fig. 6E). Together, these data underscore a correlation between the severity of neuroabnormalities in the KI brains and the extent of impaired neuronal migration and abnormal development during corticogenesis.

### The IKC signaling machinery is conserved in IKC_*R312H*_

**Fig. 7:**
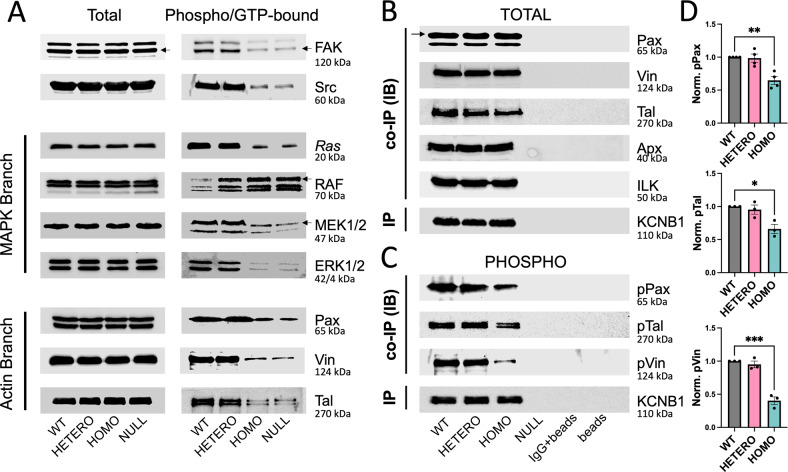
IKCs signaling is impaired in the R312H brain. **A** Representative Western blots of major components of IKC signaling (total and phosphorylated, or in the case of *Ras*, GTP-bound) from the brains of the indicated genotypes. Loading control: Bradford assay. **B** Representative co-IPs of KCNB1 (IP) with the indicated adhesome proteins. Samples were loaded to have approximately the same amount of KCNB1 protein per genotype. The same membrane was used to visualize Vinculin and Talin-1 (membrane stripping). **C** Representative co-IPs of KCNB1 (IP) with the indicated phosphorylated adhesome proteins: Paxillin phosphorylated at Tyr118 (pPax), Talin-1 phosphorylated at Ser425 (pTal), and Vinculin phosphorylated at Tyr100 (pVin). Samples were loaded to have approximately the same amount of KCNB1 protein per genotype. The same membrane was used to visualize pVinculin and pTalin-1 (membrane stripping). Controls: mouse IgG and empty beads. **D** Densitometric quantifications of 3–4 experiments as those shown in (**C**). *P < 0.05, **P < 0.01, and ***P < 0.001 (one-way ANOVA, Tukey’s post hoc).

IKCs signal through integrins, which have an established importance in determining neocortical developmental processes [12]. Therefore, we checked major components of the IKC signaling machinery (Fig. S7), by Western blot or ELISA (Fig. 7, Table 1) [36]. Notably, the amounts of phosphorylated (a proxy for activated) protein, were comparable between WT and R312H heterozygous brains, but were significantly decreased in R312H homozygous and NULL (Fig. 7A). Representative co-immunoprecipitations of KCNB1 with adhesome proteins, Paxillin, Vinculin and Talin-1, that link integrins to the actin cytoskeleton, are illustrated in Fig. 7B, C [37]. Phosphorylation of Paxillin by FAK, allows the recruitment of Talin-1 and Vinculin, whose phosphorylation, in turn, assists the assembly process [38]. R312H subunits retained the ability to interact with the adhesome proteins; however significantly lower amounts of phosphorylated protein co-immunoprecipitated with R312H in homozygous brains, compared to control (Fig. 7D). We conclude that IKC signaling is grossly impaired in homozygous and NULL brains.

**Table 1:** Data are normalized to WT.

	HETERO	HOMO	NULL	% SD WT	WT vs. HOMO	WT vs. NULL
*pFAK*	1.03 ± 0.06	0.45 ± 0.04	0.51 ± 0.11	12	<0.01	<0.01
*pSrc*	0.92 ± 0.06	0.40 ± 0.03	0.40 ± 0.09	5	<0.01	<0.05
*Ras(GTP)*	0.93 ± 0.03	0.53 ± 0.07	0.52 ± 0.10	5	<0.05	<0.05
*pRAF*	1.58 ± 0.61	2.55 ± 0.13	2.80 ± 0.61	35	NSS	<0.05
*pMEK*	0.97 ± 0.03	0.55 ± 0.03	0.41 ± 0.09	5	<0.05	<0.05
*pERK*	1.0 ± 0.1	0.39 ± 0.1	0.37 ± 0.04	9	NSS	<0.05
*pPax*	0.98 ± 0.04	0.44 ± 0.1	0.44 ± 0.09	7	<0.01	<0.01
*pTal*	0.96 ± 0.05	0.49 ± 0.15	0.45 ± 0.13	5	<0.05	<0.05
*pVin*	1.07 ± 0.06	0.35 ± 0.04	0.30 ± 0.04	16	<0.01	<0.01
*pAkt*	0.78 ± 0.03	0.48 ± 0.06	0.45 ± 0.02	9	<0.01	<0.01

*WT vs. HOMO* and *WT vs. NULL* are, respectively, the Tukeys’s ad hoc comparisons of WT vs. R312H homozygous and WT vs. NULL. *N* = 3–4 experiments/protein.

*Pax* Paxillin, *Tal* Talin-1, *Vin* Vinculin, *SD WT* is the Standard Deviation of WT = 1, expressed in percent.

### IKCs enhance cell proliferation

Overall, our findings suggested that the impaired signaling of IKC_R312H_, was the culprit for the neuromorphological abnormalities of the KI brains. To test this idea, we assessed cellular processes regulated by integrins, namely proliferation, motility and neuritogenesis, in heterologous expression systems, that provide well-established assays [2, 39, 40]. Proliferation rates in Chinese hamster ovary (CHO) cells, transfected with WT or R312H cDNA or empty pCi-neo vector (mock), up to 2 days post transfection (dpt), are illustrated in Fig. S8A. The WT channel significantly increased the rate of proliferation, compared to mock, in agreement with the work of others (20000 ± 980 cells/day vs. 7400 ± 136 cells/day, *P* = 0.017) [41, 42]. In contrast, R312H was less effective in enhancing proliferation (rate = 12500 ± 1440 cells/day, *P* = 0.049 vs. WT). Integrins regulate proliferative responses primarily via the PI3K/Akt and the Ras-MAPK pathways, which were down-activated in homozygous, and NULL brains (Fig. 7A, Table 1) [43, 44]. Therefore, we determined whether overexpressing components of these pathways (FAK, Src, RAF, Akt), could rescue proliferation. Co-transfection with R312H or mock cDNA strongly enhanced the proliferation of the cells (Fig. S8B). In contrast, co-transfection with the WT channel only moderately increased the rate of proliferation. Two enzymatically inactive mutants, K295R Src and K179M Akt, reduced the proliferation rates in all experimental conditions, while a constitutively active RAF mutant, S259A, was more effective than WT RAF in enhancing proliferation of the cells [45–47].

### IKCs affect cell motility

The motility of CHO cells transfected with WT--assessed in the wound healing assay--was increased compared to mock and R312H, in agreement with previous reports (Fig. S8C) [2, 39, 41]. However, pharmacological enhancement of the enzymatic activity of MEK, by agonist Platelet Activating Factor C-16 (PAF C-16, Fig. S8C) or FAK, by agonist Angiotensin II (Ang II, Fig. S8D), showed a trend toward accelerating wound closure in cells transfected with R312H, while had negligible, or even negative, effects on the motility of cells expressing WT or mock [48–51]. To rule out that the pharmacological agents did not alter KCNB1 current, we recorded whole-cell currents in cells incubated in the absence/presence of Ang II, following the same protocols used for cell migration. In agreement with a previous study, R312H macroscopic currents were smaller, and activated at more depolarizing voltages, compared to WT (Fig. S8E, F). We cannot rule out that the shift in the voltage-dependence was due to the presence of integrins in the complex, but this possibility seems unlikely, since the R to H replacement occurs in the voltage sensor domain) [2]. Most importantly, the recordings did not reveal any effect of Ang II on KCNB1 current.

### IKCs enhance neuritogenesis

Neurites were longer in differentiated mouse neuroblastoma N2a cells expressing WT compared to mock, in agreement with previous reports (Fig. S9A) [2, 52]. Soma hypertrophy was also noticeable in cells expressing the WT channel (Fig. S9B). Most importantly, R312H only moderately enhanced neurite outgrowth and soma hypertrophy [2]. Treatment with Ang II (Fig. S9A, B), or PAF C-16 (Fig. S9C, D), significantly boosted neurite outgrowth and soma hypertrophy in cells expressing R312H, to WT levels, but were fairly ineffective, or even negative, in the cells expressing WT or mock.

### Angiotensin II corrects morphological defects of R312H primary neurons

**Fig. 8:**
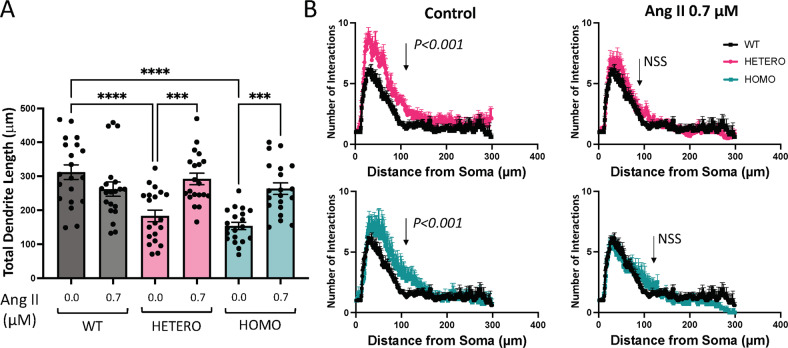
Ang II corrects morphological defects of R312H primary cortical neurons. **A** Total dendrite length of DIV14 primary cortical neurons from the indicated genotypes, incubated in the absence/presence of 0.7 μM Ang II. *N* = 20 neurons/genotype, from 4 WT pups; 5 HETERO pups and 4 HOMO pups. **B** Mean Sholl intersection profiles of the primary neurons of the various genotypes incubated in the absence presence of 0.7 μM Ang II. Each mean SIP was obtained by averaging 20 individual SIPs from 4 WT pups; 5 HETERO pups and 4 HOMO pups. *P* < 0.001 for WT vs. heterozygous R312H; *P* < 0.001 for WT vs. homozygous R312H in control and not statistically significant (NSS) in the presence of Ang II (Kolmogorov–Smirnov test). *P < 0.05*,* ***P < 0.001, and ****P < 0.0001 (one-way ANOVA, Tukey’s post hoc).

We next sought to determine whether some of the defects of native cells were recapitulated by cultured neurons, and whether impaired IKC signaling was an underlying cause. To better reproduce physiological conditions, we employed co-cultures of cortical neurons and glia, that we analyzed using Sholl analysis [53]. Thus, the TDL of heterozygous, and homozygous KCNB1+ neurons, were significantly shorter, roughly halved in the latter, compared to control (Fig. 8A). Incubation with 0.7 μM Ang II significantly increased the TDL of the heterozygotes by ∼25%, and the homozygotes by ∼50%, while had a slightly negative effect on WT neurons. Likewise for native neurons, also the SIPs of the primary neurons were broader than control SIP, reflecting dysregulated branching. Ang II treatment reversed the shape of the SIP to control (Fig. 8B).

### Angiotensin II increases the synaptic connectivity of R312H primary neurons

**Fig. 9:**
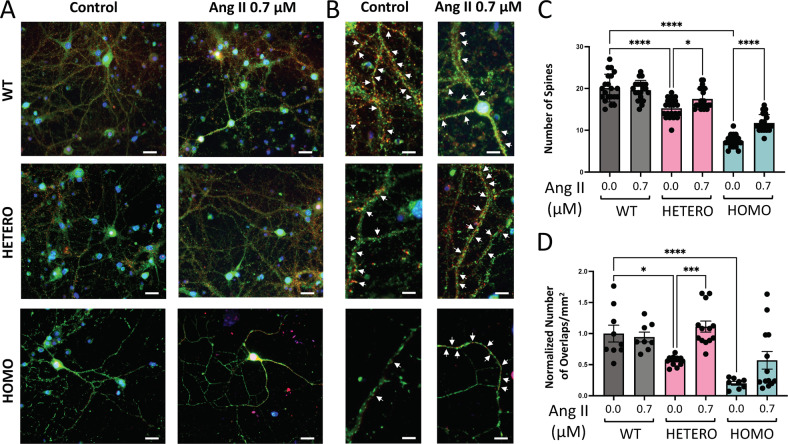
Ang II enhances synaptic connectivity of R312H primary neurons. **A** Representative images showing DIV14 primary cortical neurons of the indicated genotypes co-stained with Syn-1 (green), PSD-95 (red) to visualize functional synapses (arrows), and DAPI (blue), in control or incubated in the presence of 0.7 μM Ang II. **B** Magnifications are taken from the images on the left. Scale bars 50 μm for small magnification images and 10 μm for large magnification images. **C** Average number of dendritic spines for the cells of the indicated genotypes incubated in the absence/presence of 0.7 μM Ang II. For a single neuron, the number of spines was counted over a single dendrite for a continuous length of ∼100 μm. *N* = 20 neurons/genotype from: WT: 4 pups; HETERO: 6 pups; HOMO: 6 pups. **D** Number of functional synapses in co-cultures of primary neurons of the indicated genotypes. Cells were co-stained with SYN-1, PSD-95 and DAPI and functional synapses were identified by SYN-1 and PSD-95 signals co-localizations. *N* = WT (cnt.-Ang): 8–9 cultures; HETERO: 13–14 cultures; HOMO: 8–7 cultures obtained from: 3 WT, 5 HETERO and 4 HOMO pups. Two technical replicates/culture. Data were analyzed with ImageJ/Fiji software and normalized to WT in control conditions. *P < 0.05; ***P < 0.001, and ****P < *0.0001* (one-way ANOVA, Tukey’s post hoc).

At DIV14, both heterozygous and homozygous neurons had less dendritic spines than WT neurons. Most importantly, Ang II treatment significantly increased the number of spines in both genotypes, and had slightly negative effect on control cells (representative images in Fig. S10 and quantifications in Fig. 9C). The number of functional synapses (SYN-1/PSD-95 co-staining) was decreased in KI neurons, reflecting the fact that their dendrites had less spines than control (Fig. 9A, B, D). Following a script well established, Ang II treatment increased the number of functional synapses in heterozygous and homozygous co-cultures—in the case of the former, to WT levels—consistent with the enhancing effect of Ang II on spine maturation, and had no effect on WT co-cultures.

### Inhibiting IKC signaling causes Kcnb1^R312H^-like morphological defects in WT neurons

To confirm that impaired IKC signaling causes morphological abnormalities, we inhibited it in WT neurons. Thus, we incubated DIV14 WT neurons with Cilengitide [synonymous Cyclo(-RGDfK)], an inhibitor of integrin-α5 in the 10–100 nM range, and separately, with FAK inhibitor PND-1186, that had both shown efficacy with IKCs in vivo and in vitro [3, 54–56]. The inhibitors caused a significant decrease in the total number of mature spines (Fig. S11A, B), and synaptic connections (Fig. S11C, D), compared to control. Treated cells presented typical dysregulated branching around the soma, reflected in broader SIPs (Fig. S11E, F). In contrast, the TDL did not change upon treatment with the inhibitors (not shown), a result that was not investigated further.

In summary, *Kcnb1*^*R312H*^ primary neurons exhibited morphological defects that recapitulated those of native neurons. These abnormalities could be suppressed, or induced, by impinging on the IKC signaling machinery. Hence, these data implicate the non-ionic functions of IKCs into the mechanisms underlying the morphology of glutamatergic cortical neurons.

## Discussion

To answer fundamental questions about the role of IKCs in prenatal brain development and their associated channelopathies, we constructed a *Kcnb1* null mouse and a KI mouse harboring the *Kcnb1*^*R312H*^ gene variant, originally mapped in children with developmental and epileptic encephalopathies [57]. We report that NULL and R312H mice exhibited gross disorganization of the cortical layers, along with disrupted synaptic connectivity, frequent, spontaneous seizures, anxiety and compulsive behavior. R312H KCNB1 subunits formed complexes with α5β5 integrins, but in homozygotes the signaling of those macromolecules was grossly compromised. Notably, rescuing IKC signaling in vitro, through overexpression, or pharmacological activation of its components, suppressed the IKC_R312H_ phenotype. Overall, these results underscore a key role of IKCs in prenatal neuronal migration, that they appear to control through non-ionic functions.

The R312H KI mouse may contribute to the debate in epilepsy research, concerning the potential epileptogenic role of brain defects [58]. The ionic current appears to be the primary culprit for the severe epileptiform activity of the mice, as heterozygotes did not exhibit appreciable neuroabnormalities. In contrast, the malformations observed in homozygotes appear to be originated by impaired IKC signaling during prenatal development. Thus, these studies may provide mechanistic insight into the underlying causes of brain defects, often detected in DEE patients harboring *KCNB1* gene mutations--that were poorly understood [7, 57]. Nonetheless, the interplay between ionic and non-ionic functions is synergetic and intrinsically linked in IKCs [1, 2]. Therefore, it is likely that also IKC current plays a role in the context of neurodevelopment. In fact, many of the mechanisms underlying neurodevelopment are both hard-wired in the neurons and activity-dependent [59–61]. For example, spontaneous electrical activity influences neural circuit development and is crucial for laying out early connectivity maps in many areas of the brain [62–64]. Thus, it is possible that during the formation of the neocortex, IKCs translate the spontaneous electrical activity of emergent circuits into biochemical signals that help guiding their formation. Future investigations will elucidate the potential role of IKC ionic functions in those mechanisms. In conclusion, this study establishes integrin-K^+^ channel complexes as major players in prenatal brain development, and supports an argument that developmental channelopathies contribute to the etiology of DEEs.

## Supplementary information

supplemental figures and uncropped Western blots

uncropped Western blots

video V1-a

video V1-b

video V1-c

video V1-d

video V2 hetero

video V2 homo

video V2 WT

checklist

## Data Availability

All the data used for this study that are not presented in the figures, and any additional information required to reanalyze the data reported in this paper are available on request from the corresponding author. Mouse lines generated in this study will be deposited to the Mutant Mouse Resource & Research Centers of the NIH, upon publication of this study. This study did not generate new unique chemical reagents.
