# Supplementary material for: Integrin-KCNB1 potassium channel complexes regulate neocortical neuronal development and are implicated in epilepsy
Source: Cell Death Differ. 2022 Oct 7;30(3):687–701. doi: 10.1038/s41418-022-01072-2 (PMC9984485; doi:10.1038/s41418-022-01072-2)

SUPPLEMENTAL FIGURES

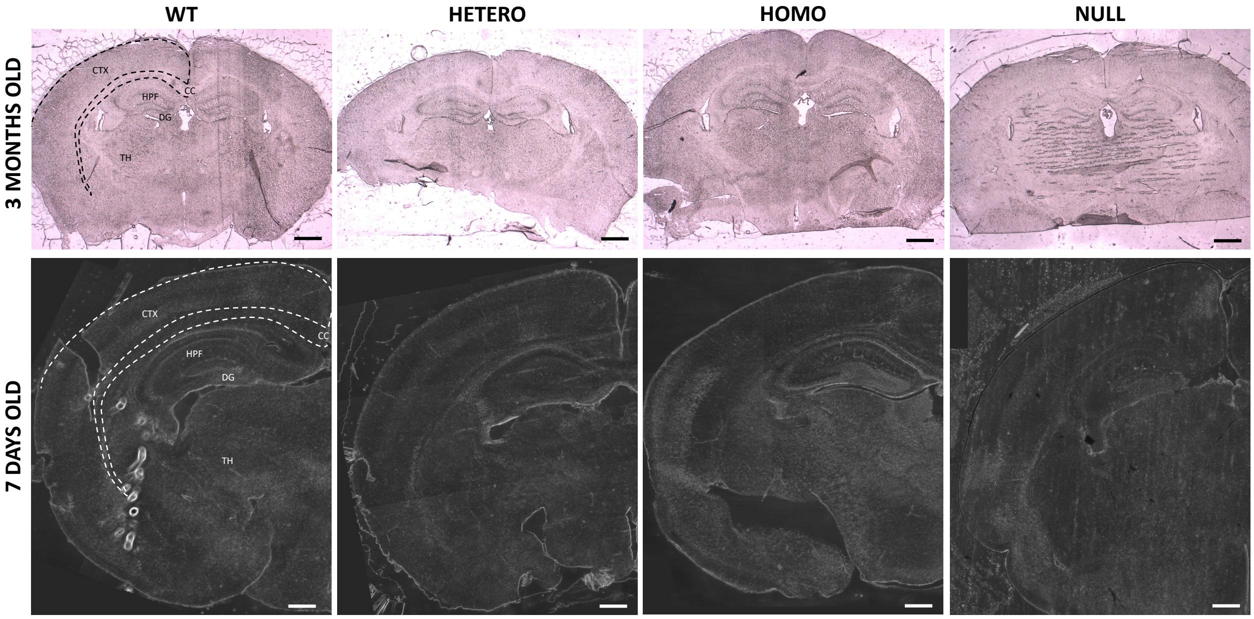

Figure S1 Bright-field images of adult and pup brain sections
A) Coronal sections of 3-month-old animals. The sections were used in the immunostainings of Fig. 2. Scale bar 1.0 mm.
B) Half coronal sections of P7 pups. The sections were used in the immunostainings of Fig. 3. Scale bar 2 mm.
The cortex (CTX) and *Corpus callosum* (CC) are highlighted by the dotted lines. Hippocampus (HPF), Thalamus (TH) and Dentate gyrus (DG) are indicated.

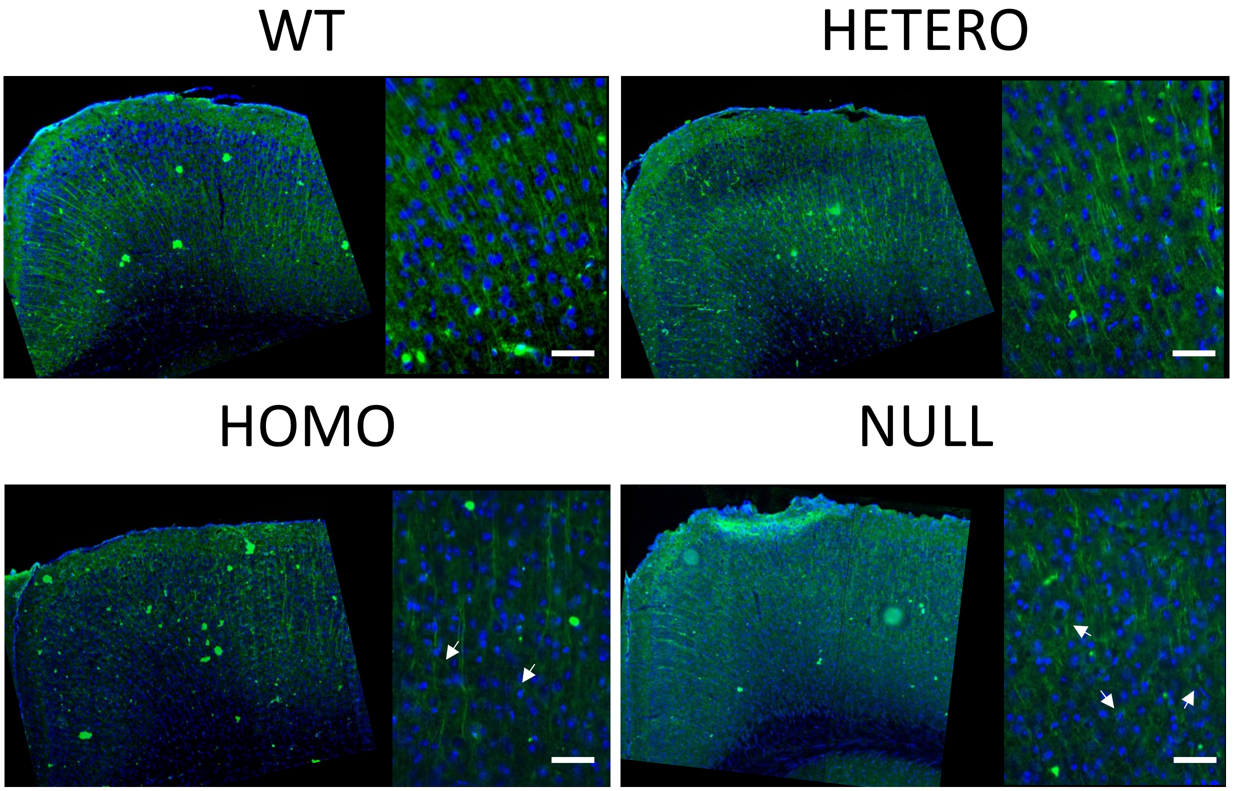

Figure S2 R312H cortices express less pyramidal neurons and exhibit neuronal disorganization

Cortical sections from 3 month-old mice of the indicated genotypes stained with Map2 (green) and DAPI (blue) at small (4x) and medium (40x) magnification. Reduced density and disrupted organization of pyramidal neurons is evident in KI cortices especially R312H homozygous and NULL (arrows). Scale bars 250 (4x) and 50 (40x) μm.

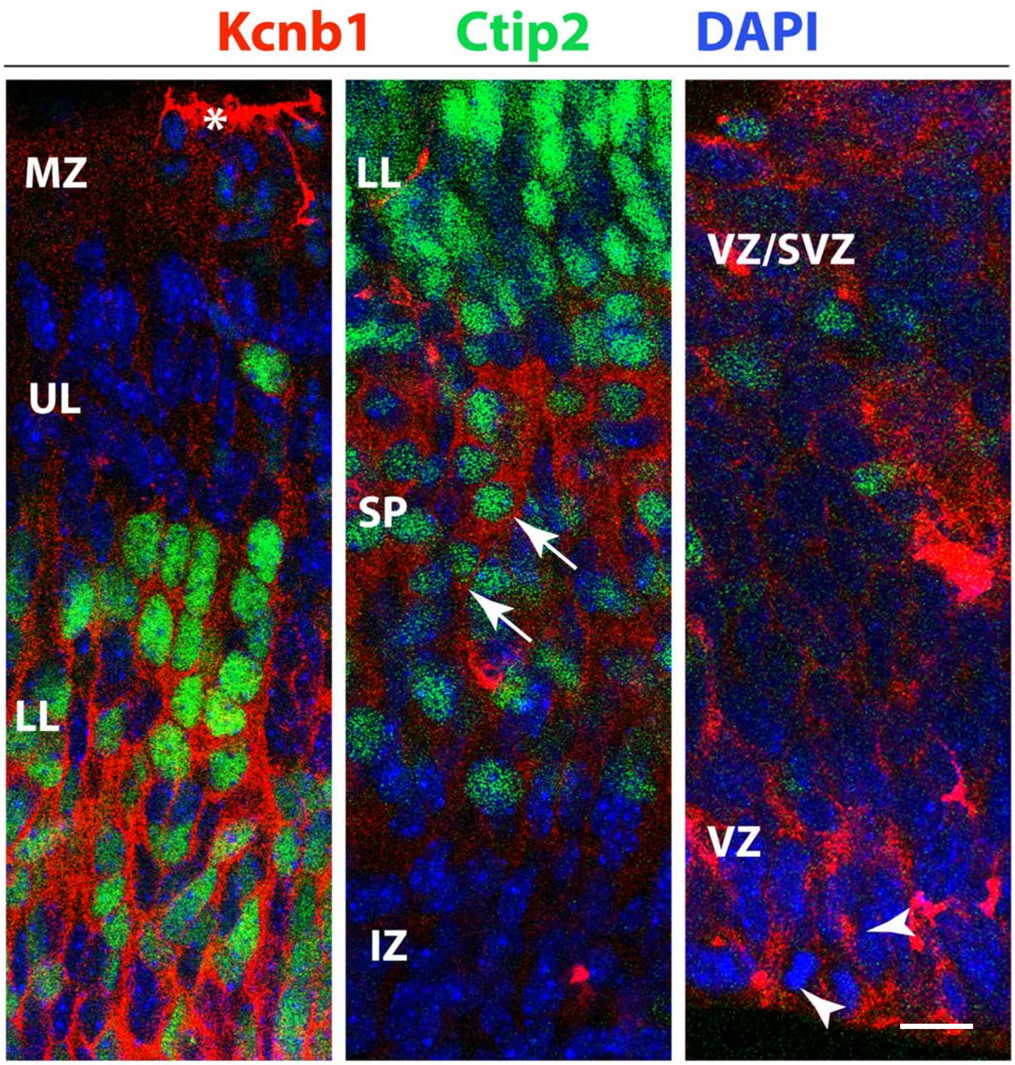

Figure S3 KCNB1 in embryonically expressed in neocortex
Representative confocal images of E17 neocortex sections immunostained for KCNB1 (red), Ctip2 (green) and DAPI (blue). KCNB1 immunoreactivity was detected in Cajal Retzius neurons of the marginal zone (MZ) and subplate (SP) neurons. In addition, KCNB1 immunoreactivity was observed in the MZ where basal end feet of radial glia terminate, within the lower and upper layers (UL) of developing cortical plate, and in the dividing radial glia at the VZ surface. Scale bar 25 μm.

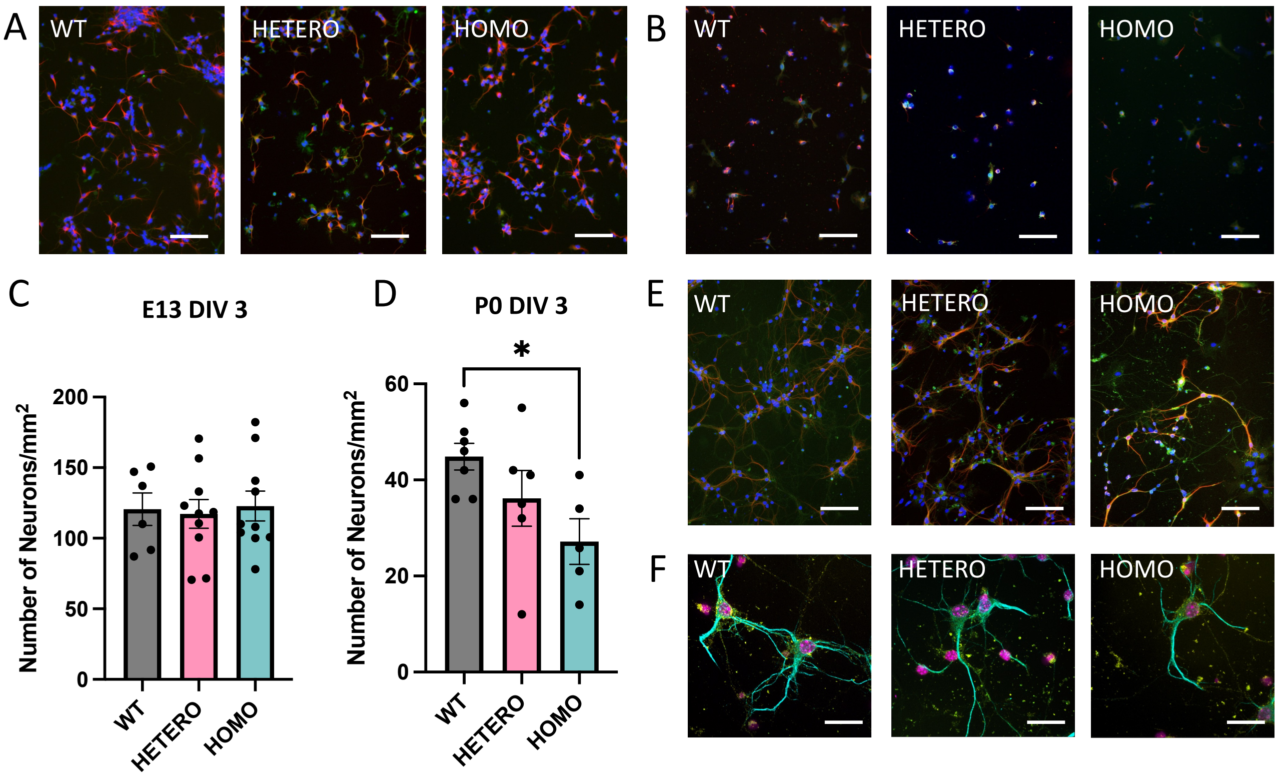

Figure S4 R312H neurons die during corticogenesis

A) Representative images of pure cultures from the various genotypes at DIV3. Neurons were co-stained with KCNB1 (green) and Map2 (red) antibody. DAPI blue color. Scale bar 100 μm.

B) As in (A) for co-cultures. Scale bar 100 μm.

C) Density of KCNB1+ neurons in pure cultures from the indicated genotypes at DIV3. WT: 6 embryos; HETERO: 10 embryos; HOMO: 10 embryos from 2-3 mice/genotype. Two technical replicates/embryo.

D) As in (C) for co-cultures. WT: 7 pups; HETERO: 6 pups; HOMO: 6 pups from 3-5 mice/genotype. Two technical replicates/pup.

E) Representative images of co-cultures for the indicated genotypes at DIV14. Map2: red; KCNB1: green and DAPI: blue. Scale bar 100 μm.

F) Representative confocal images of single neurons of the indicated genotypes. Typical KCNB1 clusters are visible in the images. Map2: cyan; KCNB1: yellow and DAPI: magenta. Scale bar 50 μm.

$*P<0.05$ (one-way ANOVA, Tukey's post hoc).

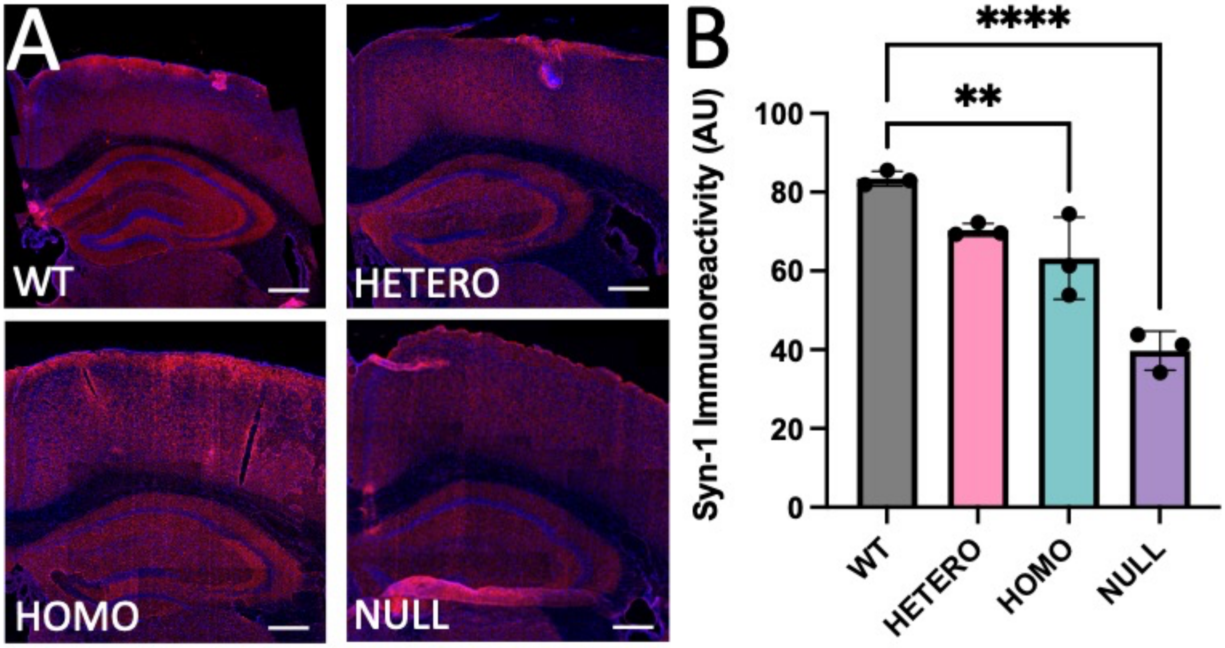

Figure S5 Connectivity is hindered in the R312H brains
A) Representative images of SYN-1 staining (red) in coronal sections cut from the brains of 3 month-old animals of the indicated genotypes. DAPI blue color. Scale bar 1 mm.
B) Quantification of the immunoreactivity to SYN-1 in IF stainings as shown in (A). N= 3 brains/genotype. Data were analyzed with Fiji **(**colors converted to 16 bits).
***P<0.01* and *****P<0.0001* (one-way ANOVA, Tukey's post hoc).

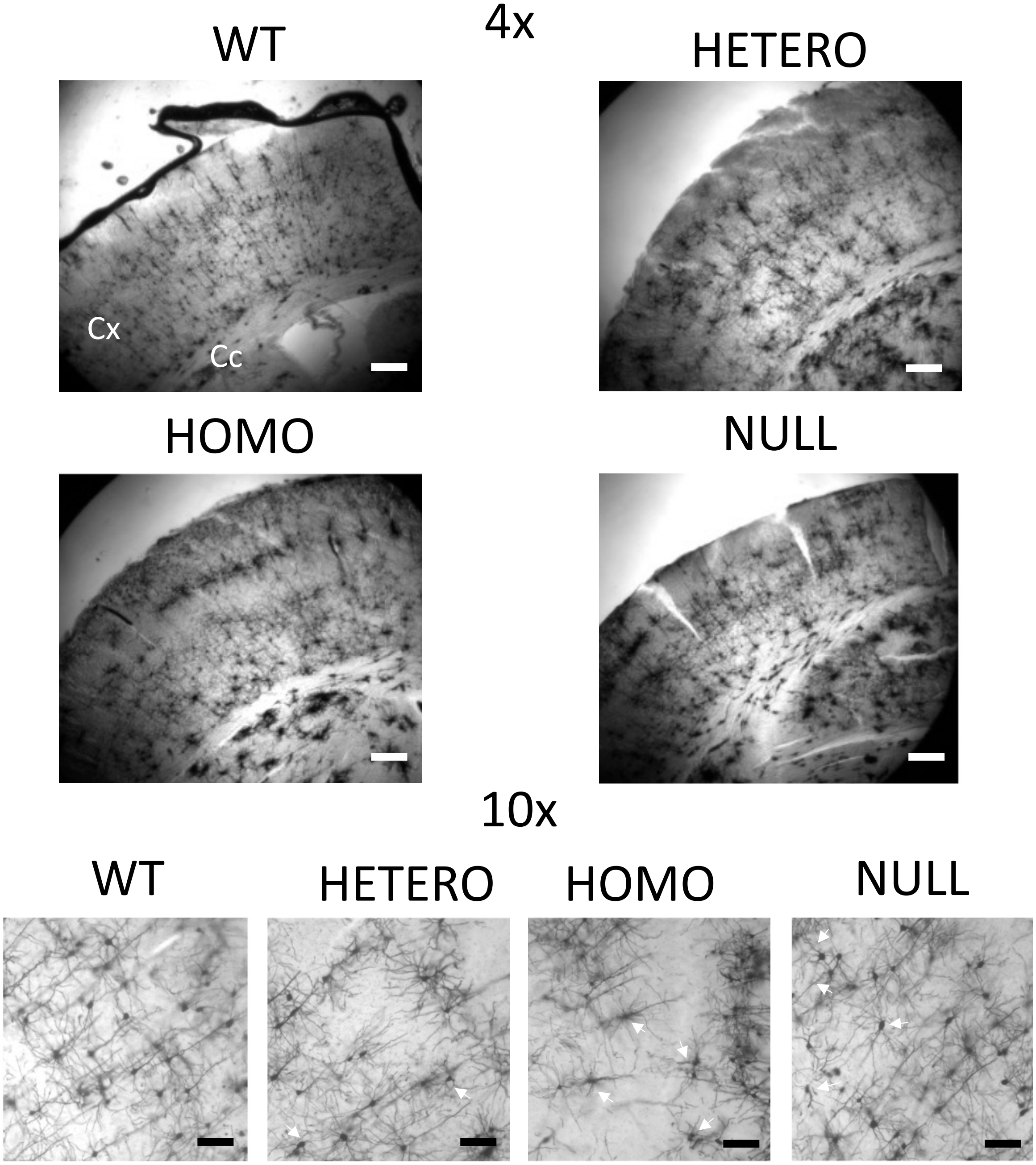

Figure S6 R312H brains show severe neuroabnormalities
Representative images of Golgi staining at 4x and 10x magnifications in lateral and central cortices of 3 month-old animals demonstrating disrupted columnar organization of apical pyramidal neurons in the KI brains. Scale bars 150 (4x) and 50 (10x) μm.

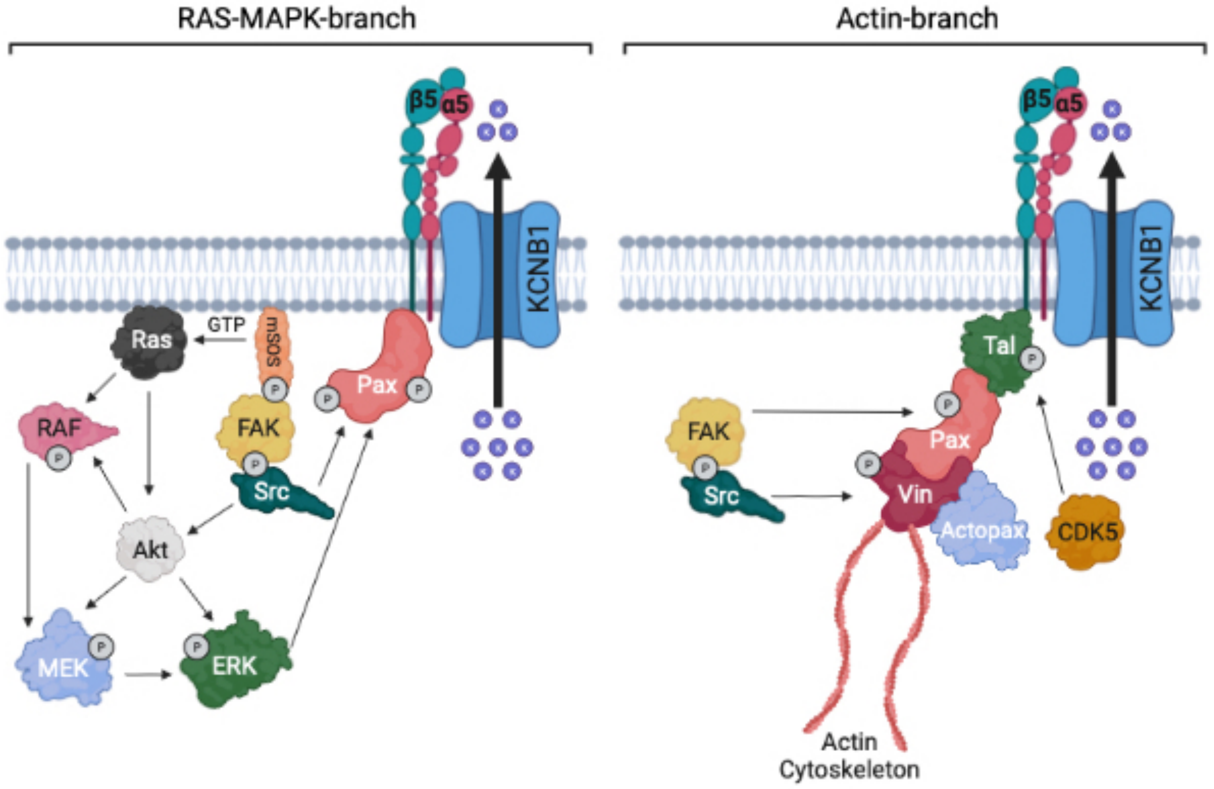

Figure S7 Proposed model of IKC signaling. Opening of KCNB1 in the IKC leads to FAK recruitment by integrins α5β5. FAK autophosphorylates Tyr397 creating a binding site for Src kinases. FAK-Src complexes then phosphorylate Paxillin at Tyr118 and Tyr31, which then bridges IKCs to *Ras*-MAPK cascades and to the actin cytoskeleton. In the *Ras*-MAPK branch, Src phosphorylates FAK at Tyr925, creating a binding site for *Ras* guanosine-triphosphate exchange factor mSOS. Following *Ras* activation, RAF is recruited to the cell membrane through binding to the switch I domain of *Ras,* and also by lipid binding. Active RAF stimulates a signaling cascade by phosphorylating MEK, which successively phosphorylates and activates downstream ERK. *Ras* and FAK-Src also interact with PI3/Akt, which regulates RAF via phosphorylation of inhibitory Ser259 and MEK and ERK signaling. Sustained *Ras*/ERK activity promotes further phosphorylation of Paxillin at Ser126.

In the actin branch, integrins interact with the adhesome, a cytoskeletal and signaling complex that promotes the assembly of actin filaments. In that branch, Paxillin recruits Vinculin, Talin-1 and Actopaxin, which link the cytoplasmic tail of integrin-β5 to actin. The assembly of Vinculin and Talin-1 is facilitated by their phosphorylation by FAK-Src and CDK5 respectively.

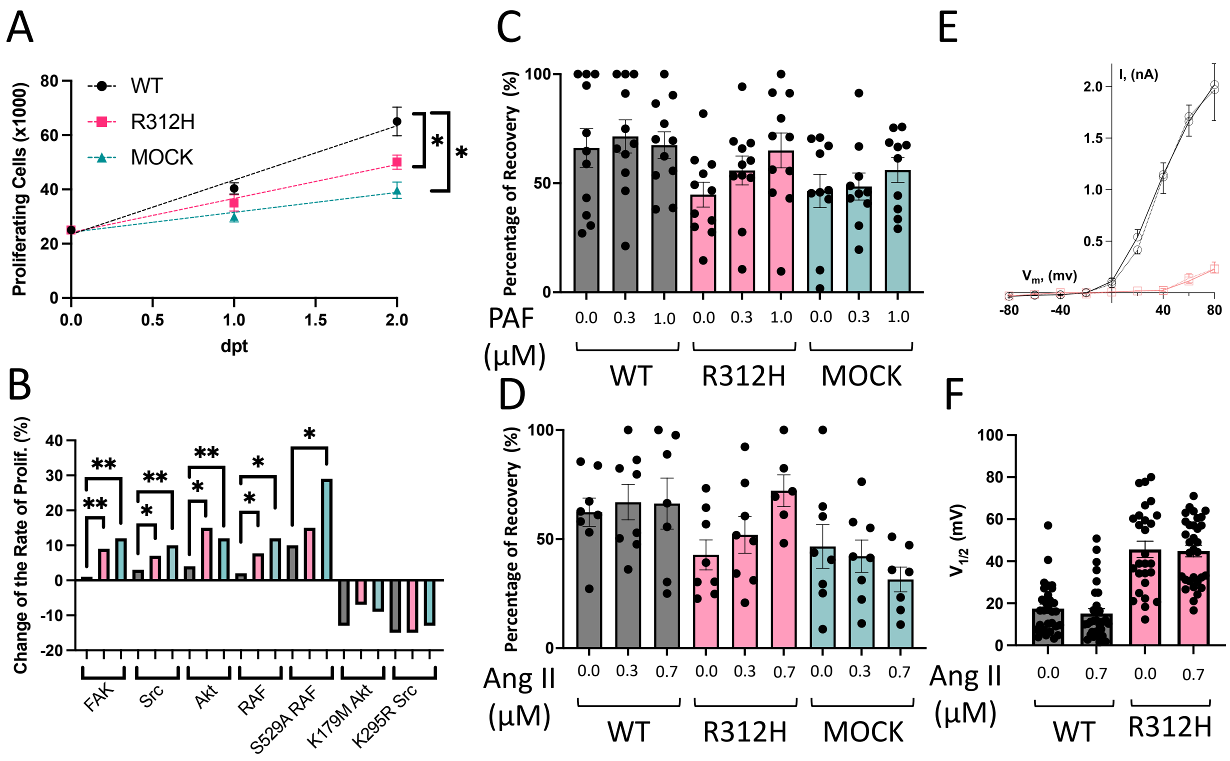

Figure S8 Rescuing IKC$_{R312H}$ signaling enhances CHO cells proliferation and migration

A) Proliferating cells vs day-post-transfection (dpt), for CHO cells transfected with WT, R312H or mock. Linear fit of the data provided proliferation rates of 20000±980; 12500±1440 and 7400±170 cells/day for WT, R312H and mock respectively. N=3 biological experiments/experimental condition with 2 technical replicates/biological experiment. **P<0.05*, (regression analysis).

B) Percent variation in proliferation rate compared to control for cells co-transfected with cDNA encoding the indicated proteins. K295R Src and K179M Akt encode enzymatically inactive kinases. In contrast, S259A RAF encodes constitutively active RAF. N=3 biological experiments/experimental condition with 2 technical replicates/biological experiment. **P<0.05*, (regression analysis).

C) Mean distance covered in 24 hours expressed as percent of the original distance (gap@24, eqn. 1) in CHO cells transfected with the indicated cDNAs in the absence/presence of PAF C-16 at the indicated concentrations. N=11 biological experiments/experimental condition. Two technical replicates/biological experiment.

D) As in (C) for cells treated in the absence/presence of Ang II at the indicated concentrations. N=7-9 biological experiments/experimental condition. Two technical replicates/biological experiment.

E) Current-voltage relationships for WT (circles) or R312H (squares) in CHO cells in control (red color) or incubated with 0.7 µM Ang II (blue color). N=40 cells/experimental condition.

F) Half-maximal voltage for activation ($V_{1/2}$) of the normalized macroscopic steady-state conductance-V relationships (G/G_{Max}, eqn. 2), were calculated by fitting the data to the Boltzmann function (eqn. 3). N=40 cells/experimental condition.

**P<0.05* and ***P<0.01* (one-way ANOVA, Tukey's post hoc).

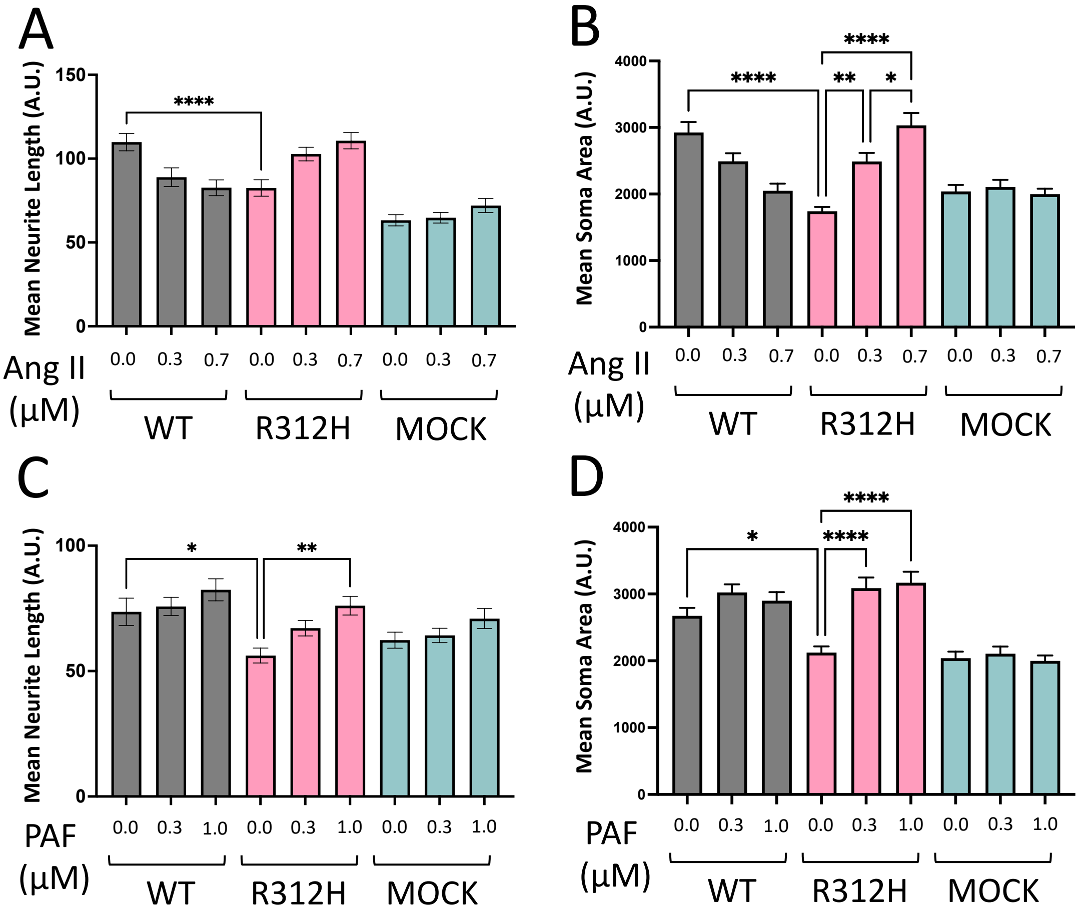

Figure S9 Rescuing IKC$_{R312H}$ signaling enhances N2a neurite outgrow

A) Mean neurite length in arbitrary units (A.U.) of N2a cells transfected with the indicated cDNAs in the absence/presence of Ang II at the indicated concentrations.

B) Mean soma area of N2a cells transfected with the indicated cDNAs in the absence/presence of Ang II at the indicated concentrations.

C) Mean neurite length of N2a cells transfected with the indicated cDNAs in the absence/presence of PAF C-16 at the indicated concentrations.

B) Mean soma area of N2a cells transfected with the indicated cDNAs in the absence/presence of PAF C-16 at the indicated concentrations.

To mark cells expressing KCNB1 WT or mutants, the cells were co-transfected with GFP and photographed 48 hours post-transfection. In all cases N=200-250 cells/experimental condition. **P<0.05, **P<0.01* and *****P<0.0001* (one-way ANOVA, Tukey's post hoc).

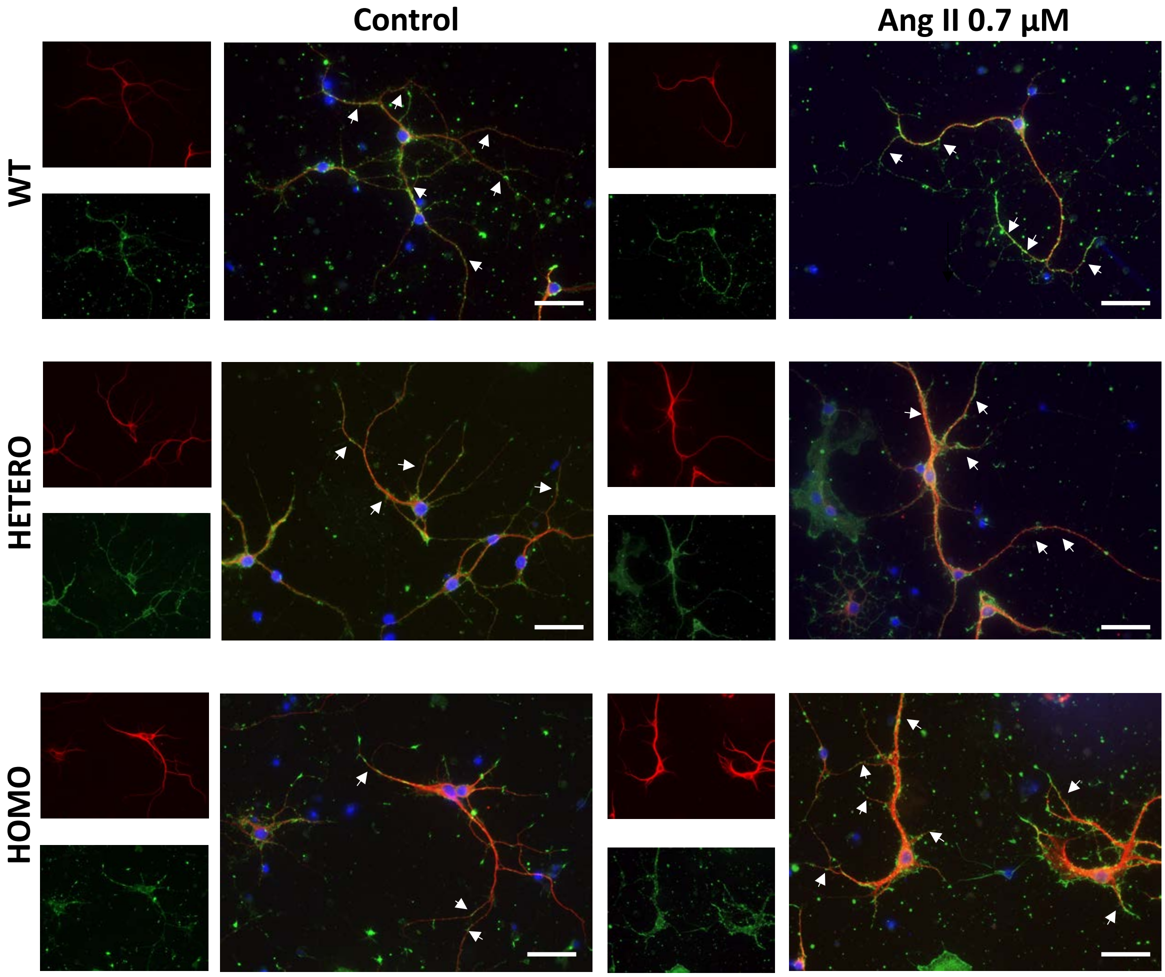

Figure S10 Angiotensin II enhances spine maturation in KI neurons
Representative images showing DIV14 primary cortical neurons of the indicated genotypes co-stained with Map2 (red), actin (green) to visualize dendritic spines (arrows), and DAPI (blue), in control or incubated in the presence of 0.7 μM Ang II. Small images: Map2 (red) and actin (green) visualizations. Large images: Map2, actin and DAPI overlaps. Scale bar 50 μm.

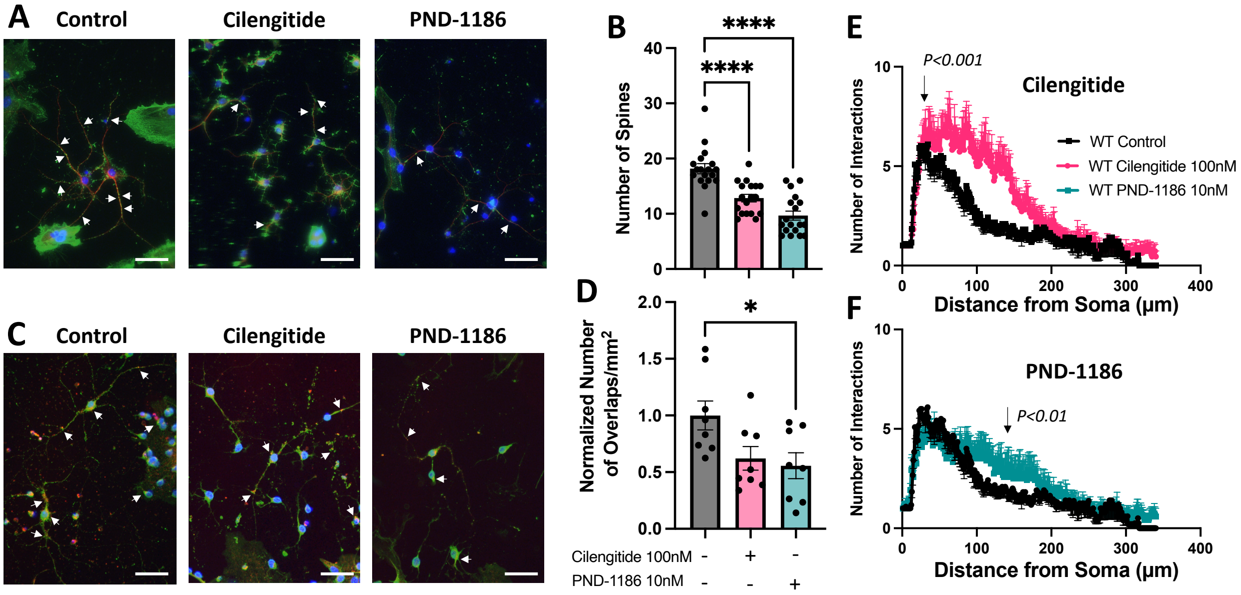

Figure S11 Cilengitide and PND-1186 cause morphological defects in WT neurons
A) Representative images showing DIV14 primary WT cortical neurons co-stained with Map2 (red), actin (green) to visualize dendritic spines (arrows), and DAPI (blue), in control or incubated in the presence of 100 nM Cilengitide or 10 nM PND-1186. Scale bar 50 μm.
B) Average number of dendritic spines for the WT cells incubated in the absence/presence of 100 nM Cilengitide or 10 nM PND-1186. For a single neuron, the number of spines was counted over a single dendrite for a continuous length of ~100 μm. N=20 neurons/genotype/experimental condition.
C) Representative images showing DIV14 primary WT cortical neurons co-stained with Syn-1 (green), PSD-95 (red) to visualize functional synapses (arrows), and DAPI (blue), in control or incubated in the presence of 100 nM Cilengitide or 10 nM PND-1186. Scale bars 50 μm.
D) Number of functional synapses in co-cultures of WT primary neurons. Functional synapses were identified by SYN-1 and PSD-95 co-localizations. N= 8 pups from 2 cultures. Two technical replicates/pup. Data were analyzed with ImageJ/Fiji software and normalized to control conditions.
E-F) Mean Sholl intersection profiles of the primary WT neurons incubated in the absence presence of 100 nM Cilengitide (E) or 10 nM PND-1186 (F). Each SIP was obtained by averaging 20 individual SIPs from 8 pups. *P<0.001* for WT vs Cilengitide and P<0.01 for WT vs PND-1186 (Kolmogorov-Smirnov test). **P<0.05, ***P<0.001* and *****P<0.0001* (one-way ANOVA, Tukey's post hoc).

UNCROPPED WESTERN BLOTS

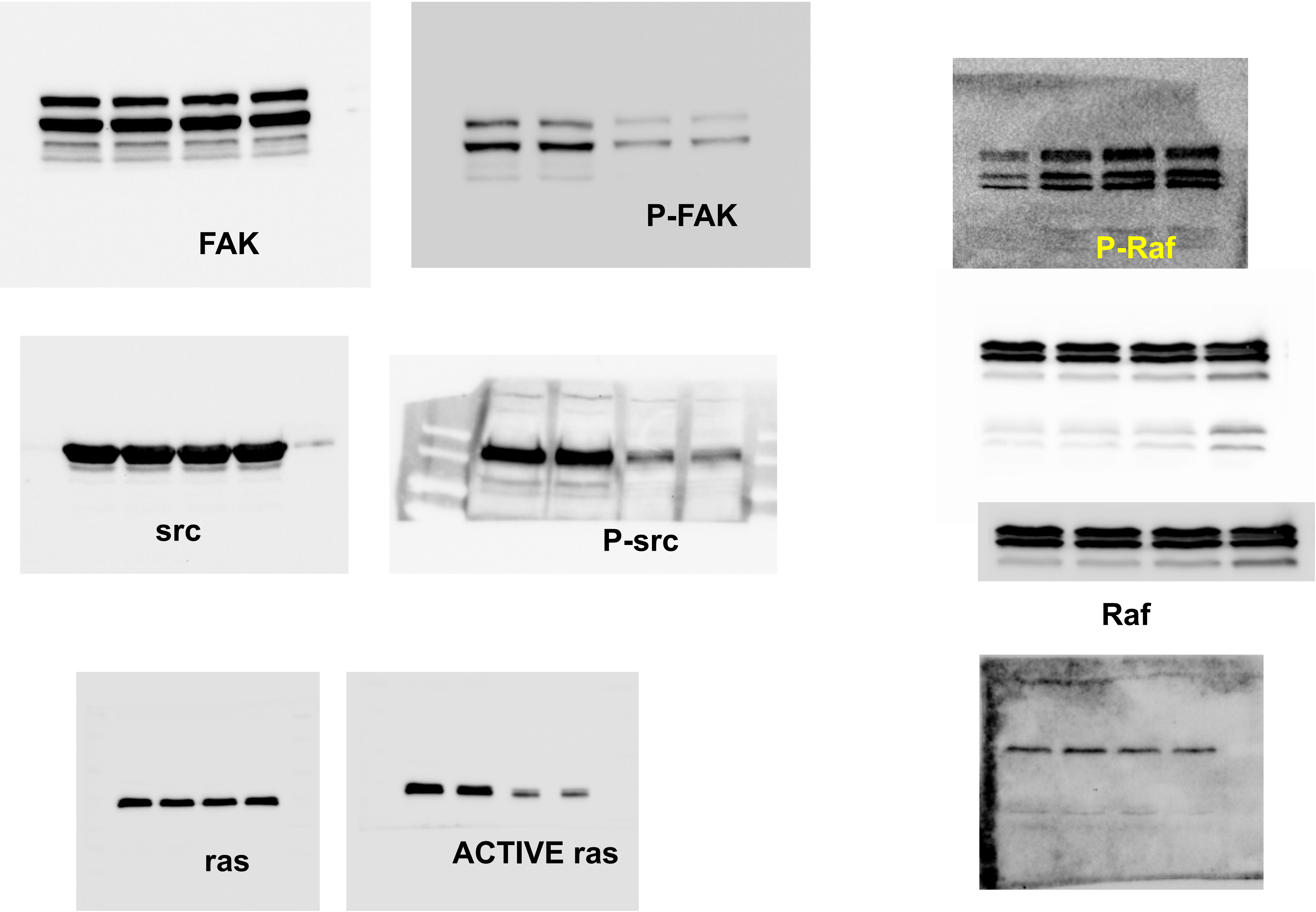

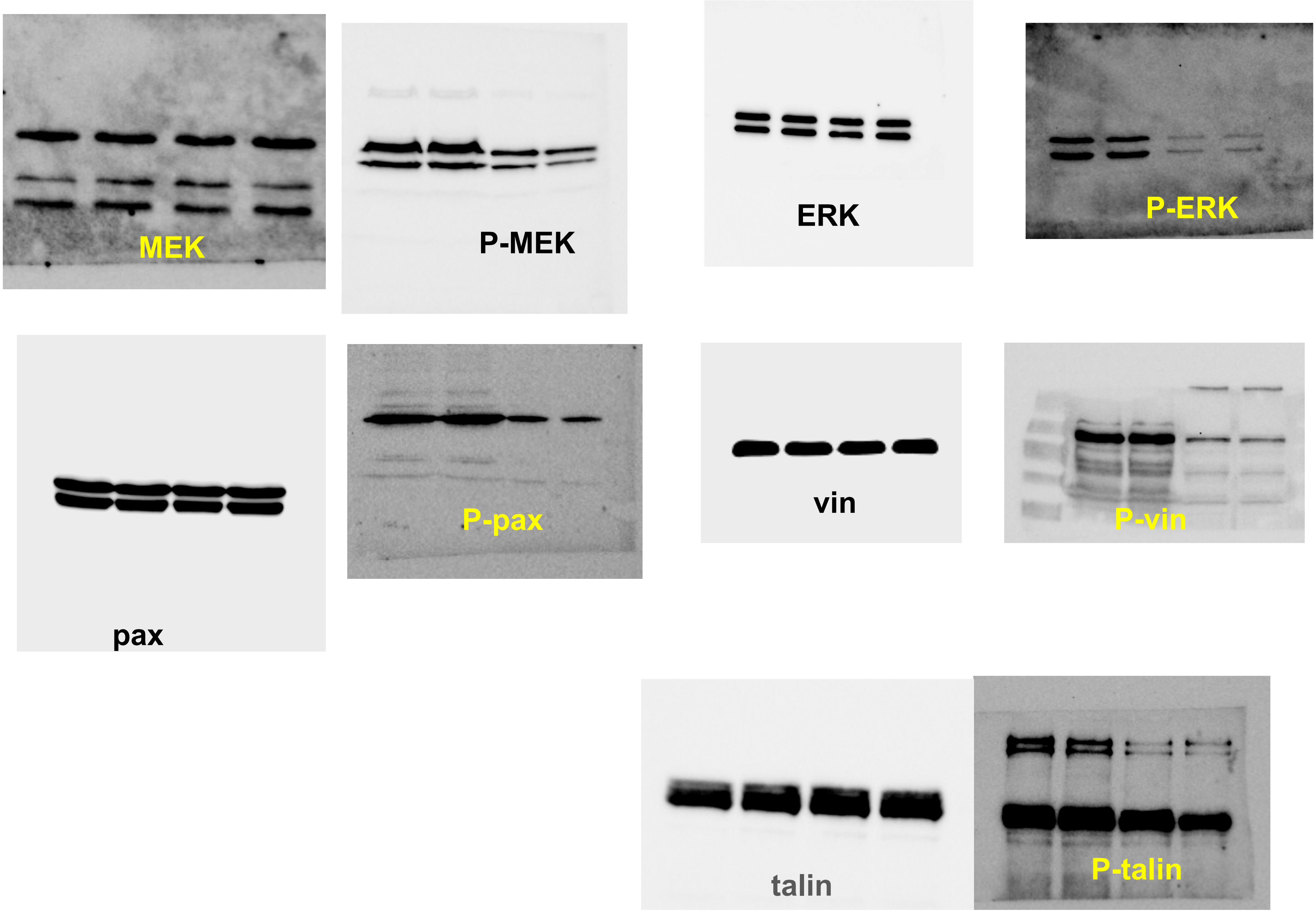
MEK
P-MEK
ERK
P-ERK
pax
P-pax
vin
P-vin
talin
P-talin

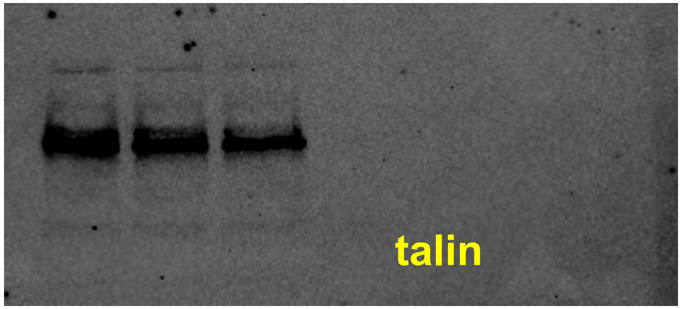
talin

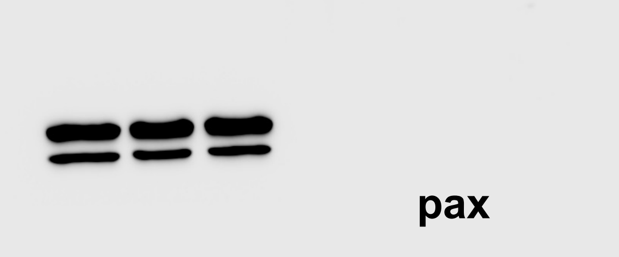
pax

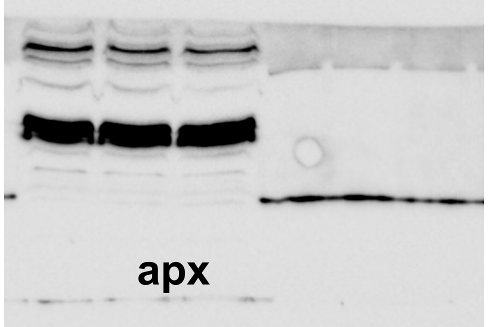
apx

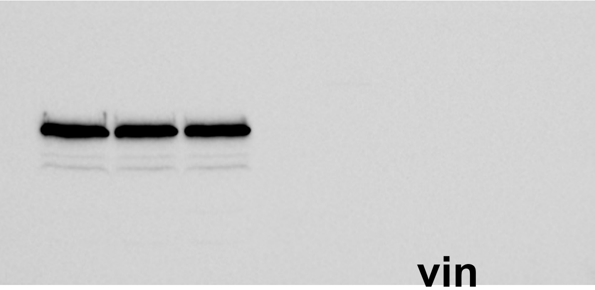
vin

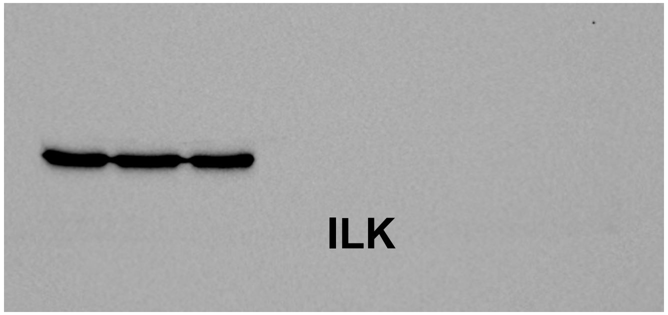
ILK

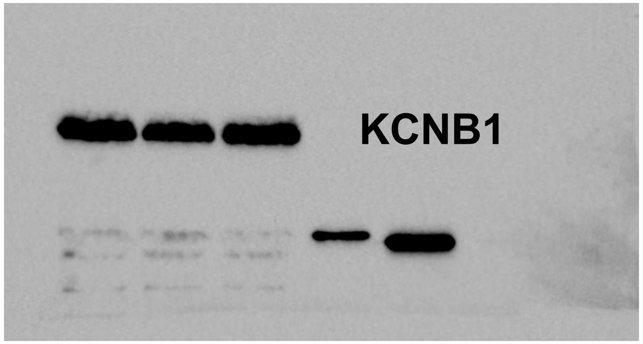
KCNB1

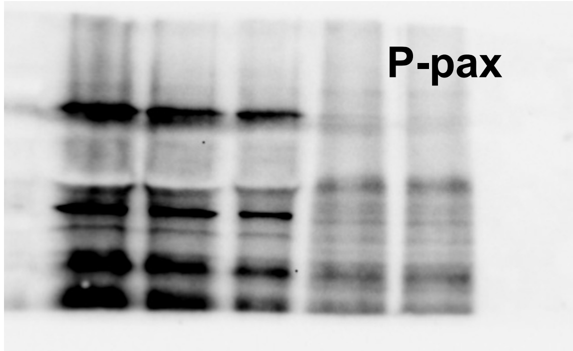
P-pax

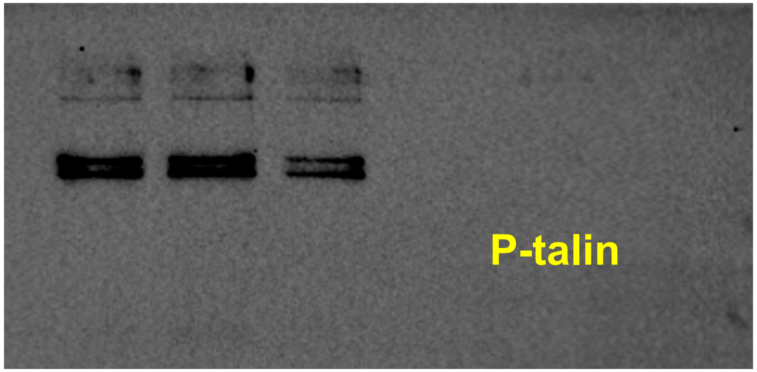
P-talin

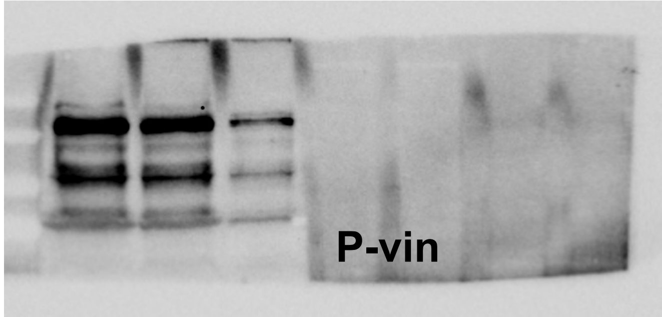
P-vin

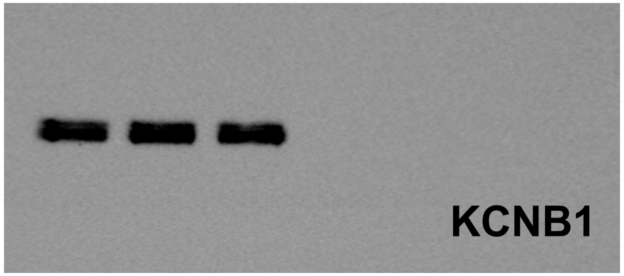
KCNB1

Fig 2

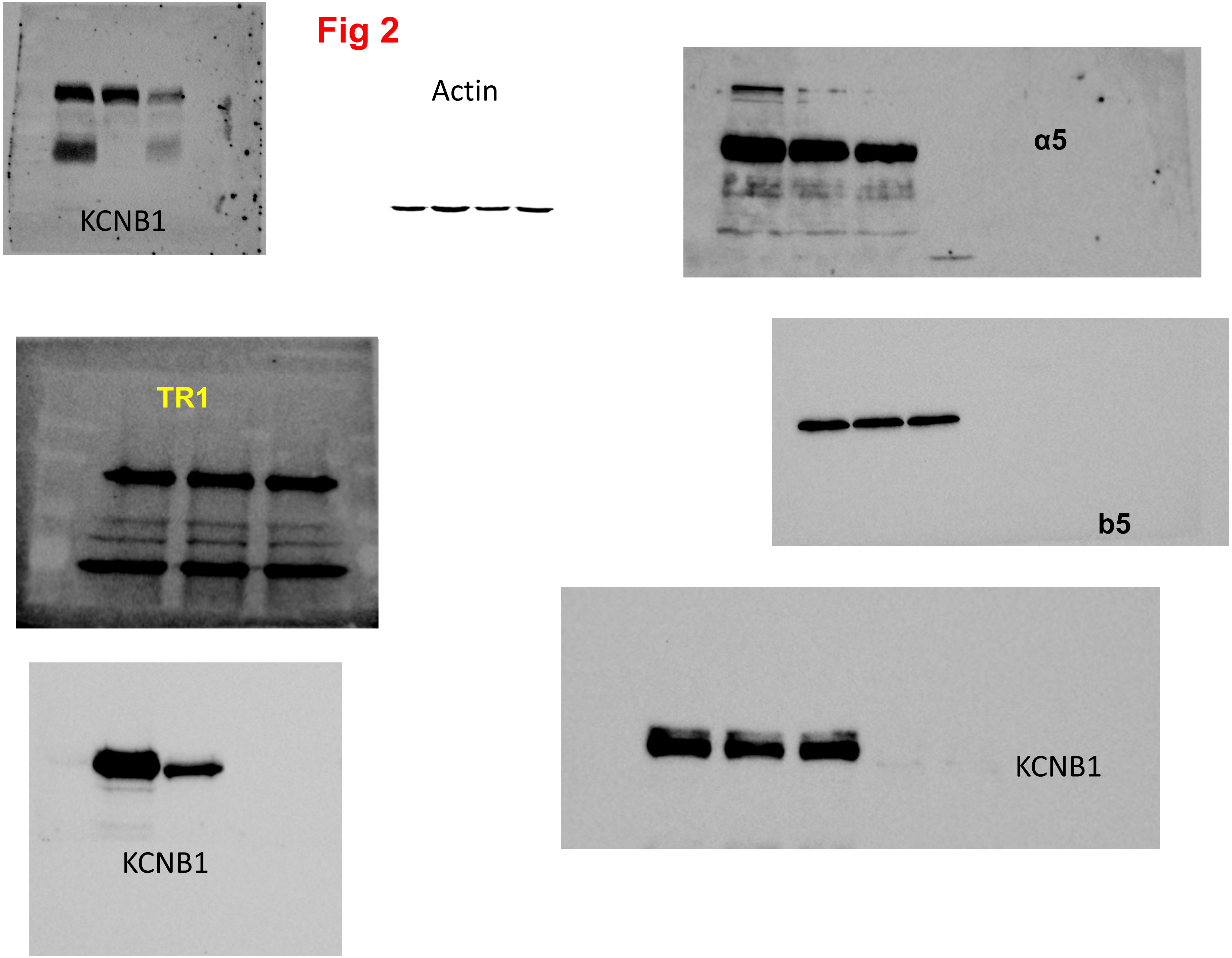

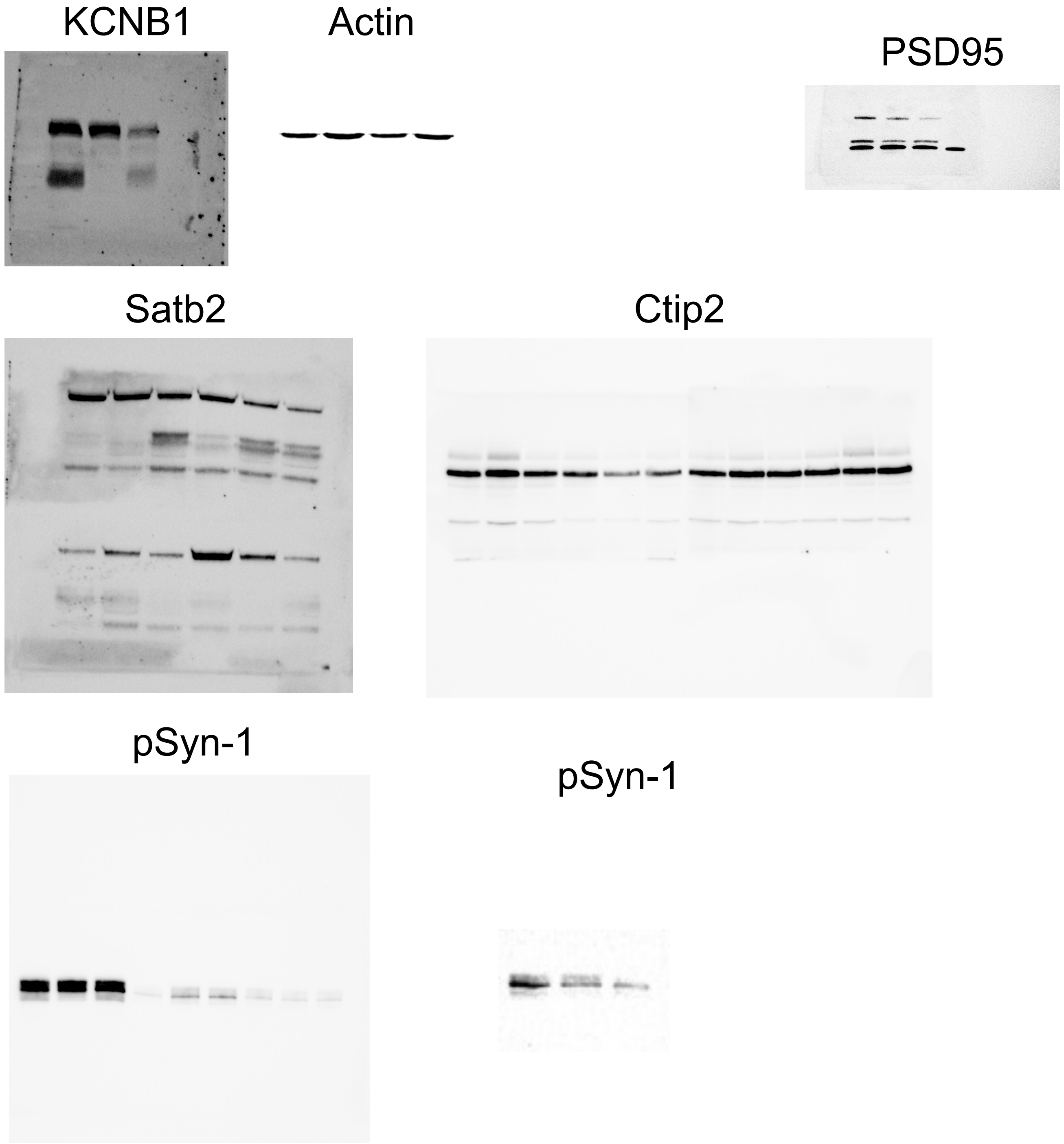
KCNB1
Actin
PSD95
Satb2
Ctip2
pSyn-1
pSyn-1

PSD95

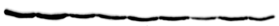

Both WTx3 Homo x3 Null x3

Syn-1

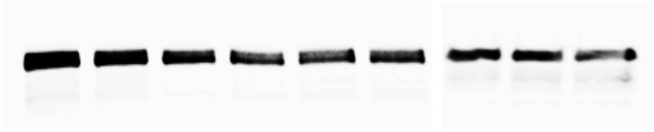

Actin

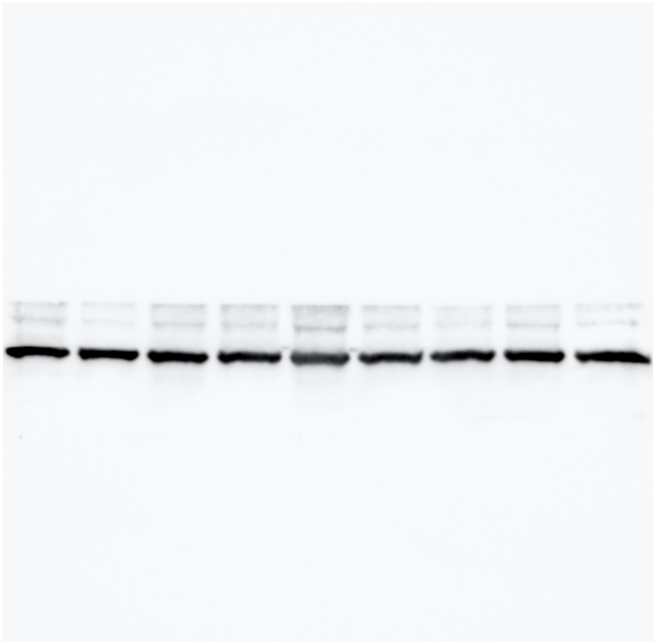

Syn-1

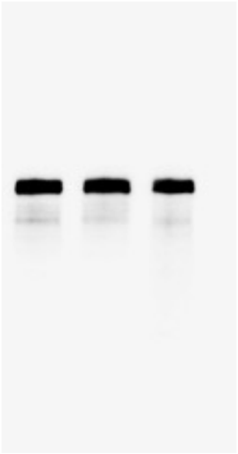

Actin

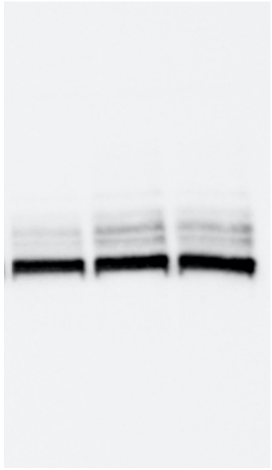

Supplement: Supplementary file 1 — supplemental figures and uncropped Western blots [file 41418_2022_1072_MOESM1_ESM.pdf]
