# Supplementary material for: Integrin-KCNB1 potassium channel complexes regulate neocortical neuronal development and are implicated in epilepsy
Source: Cell Death Differ. 2022 Oct 7;30(3):687–701. doi: 10.1038/s41418-022-01072-2 (PMC9984485; doi:10.1038/s41418-022-01072-2)

UNCROPPED WESTERN BLOTS

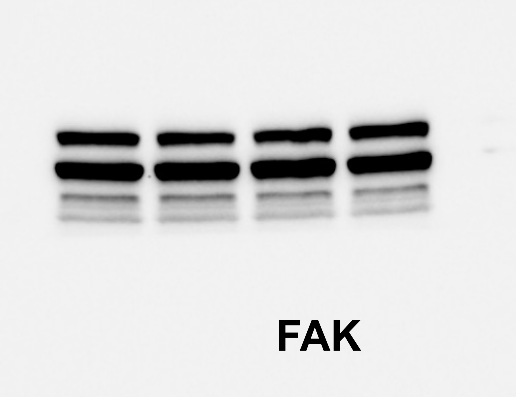

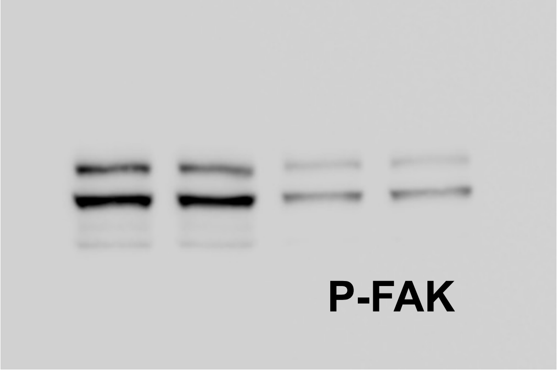

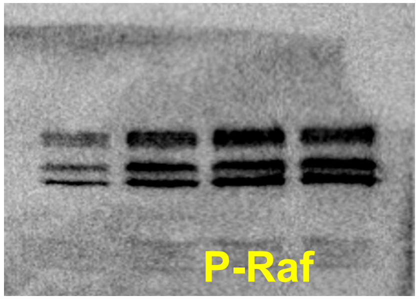

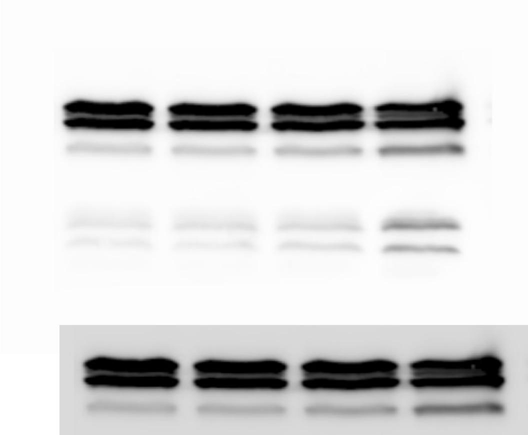

Raf

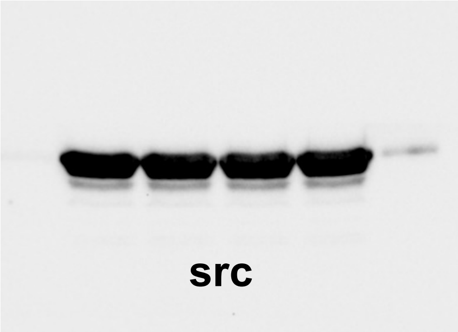

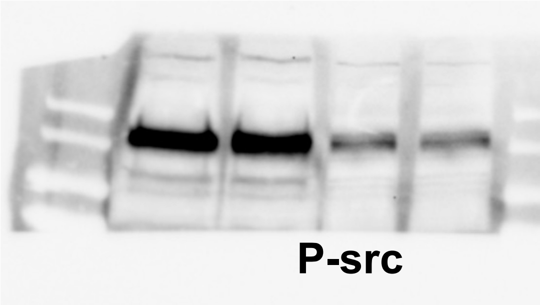

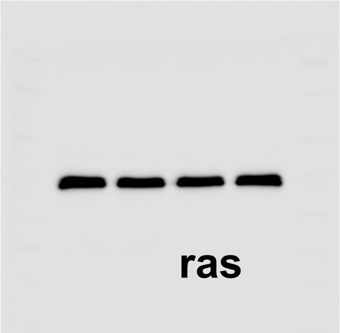

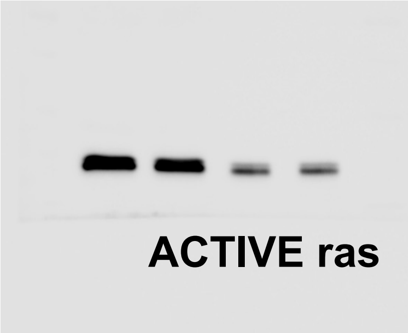

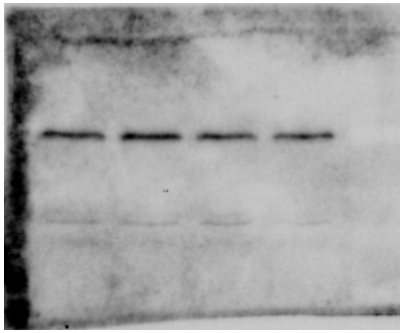

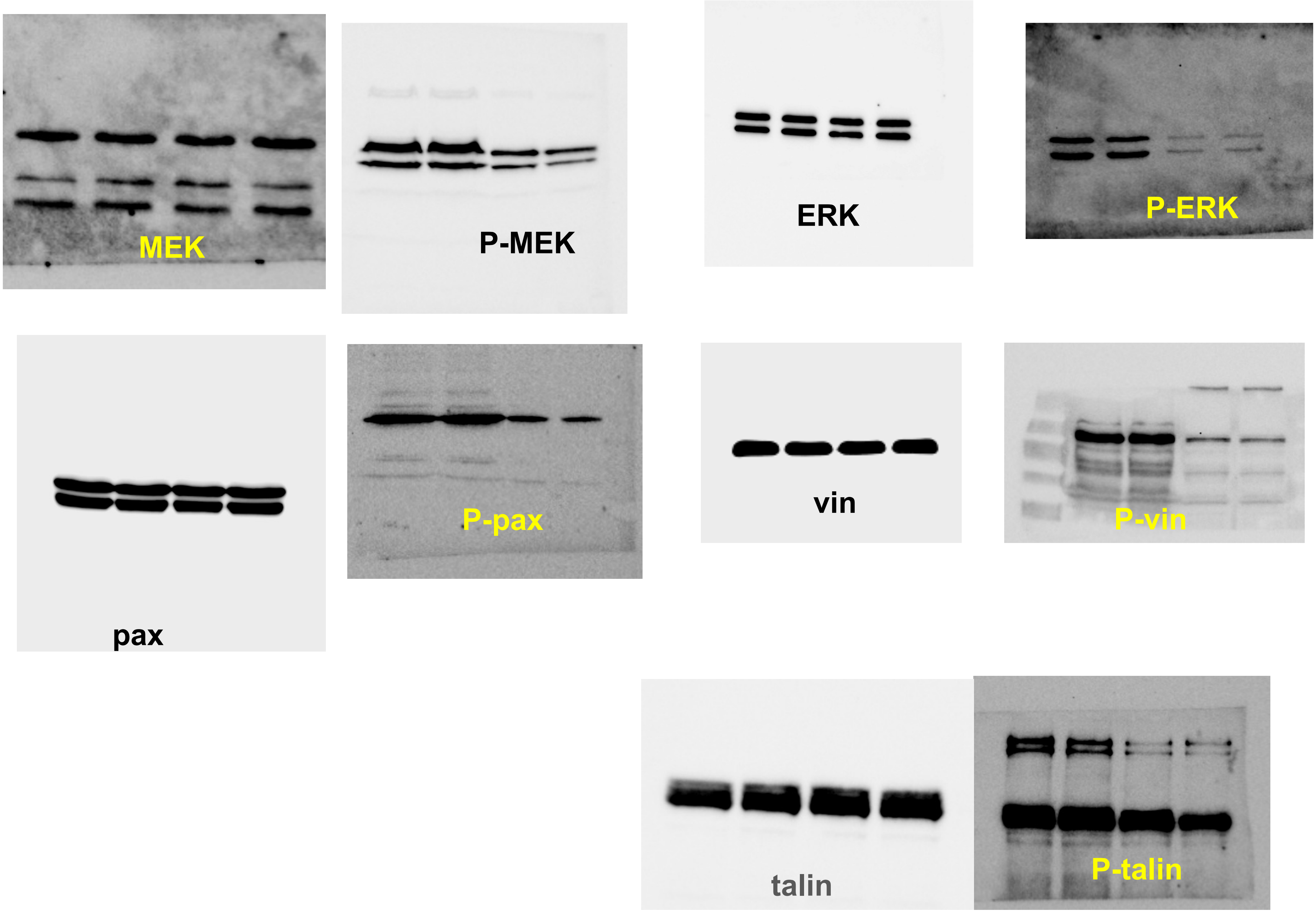
MEK
P-MEK
ERK
P-ERK
pax
P-pax
vin
P-vin
talin
P-talin

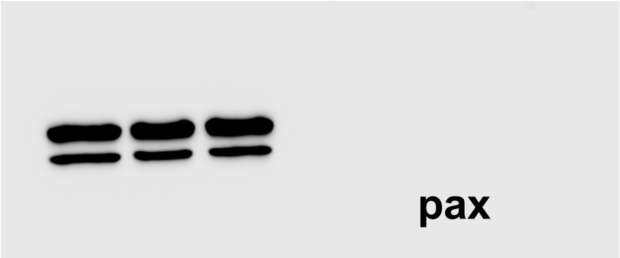
pax

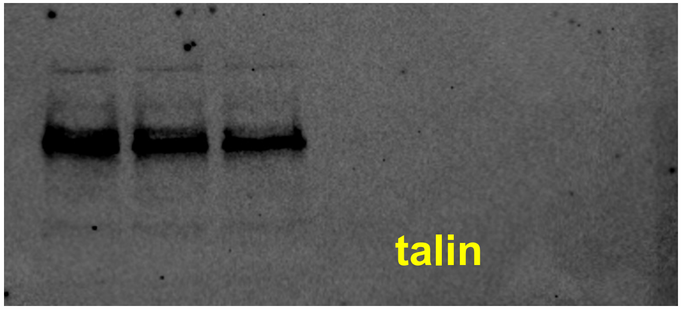
talin

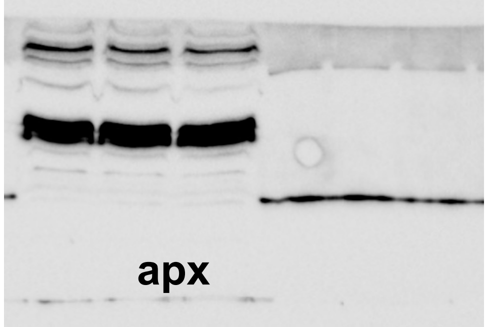
apx

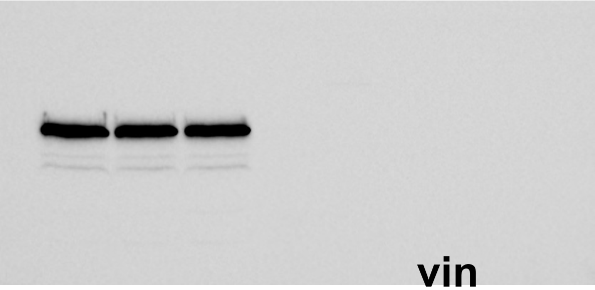
vin

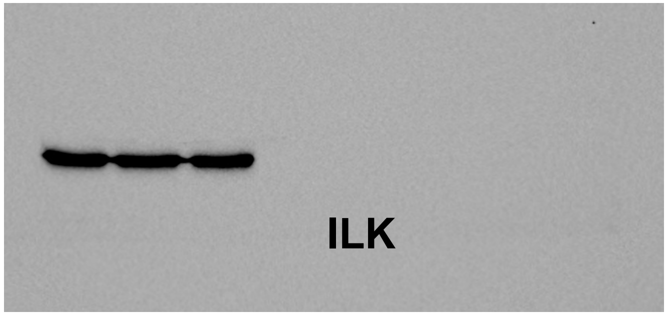
ILK

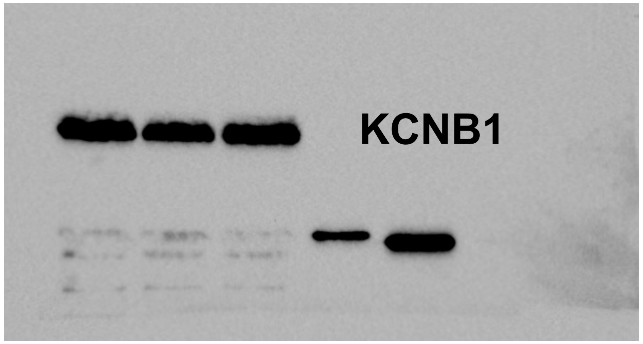
KCNB1

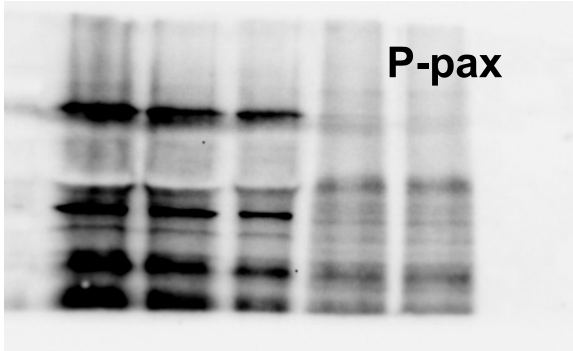
P-pax

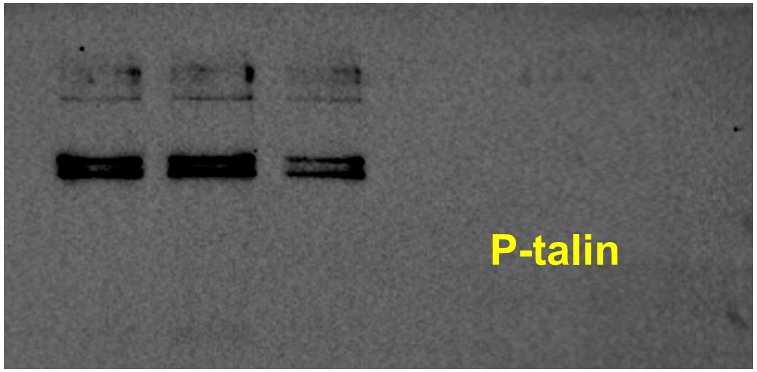
P-talin

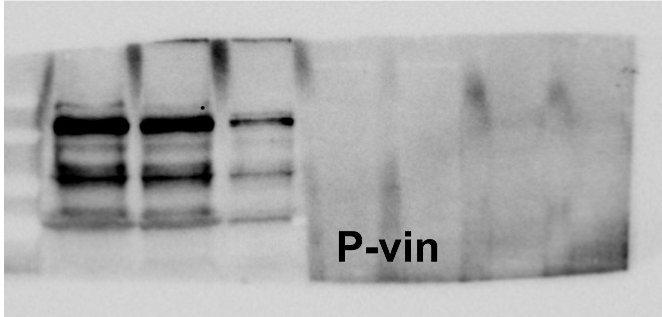
P-vin

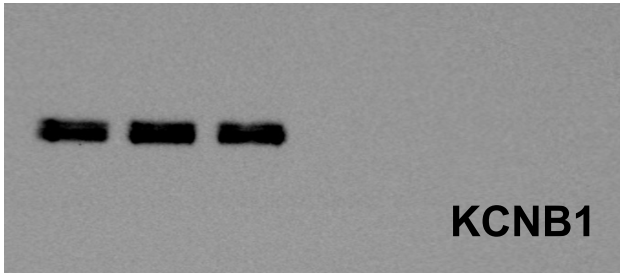
KCNB1

Fig 2

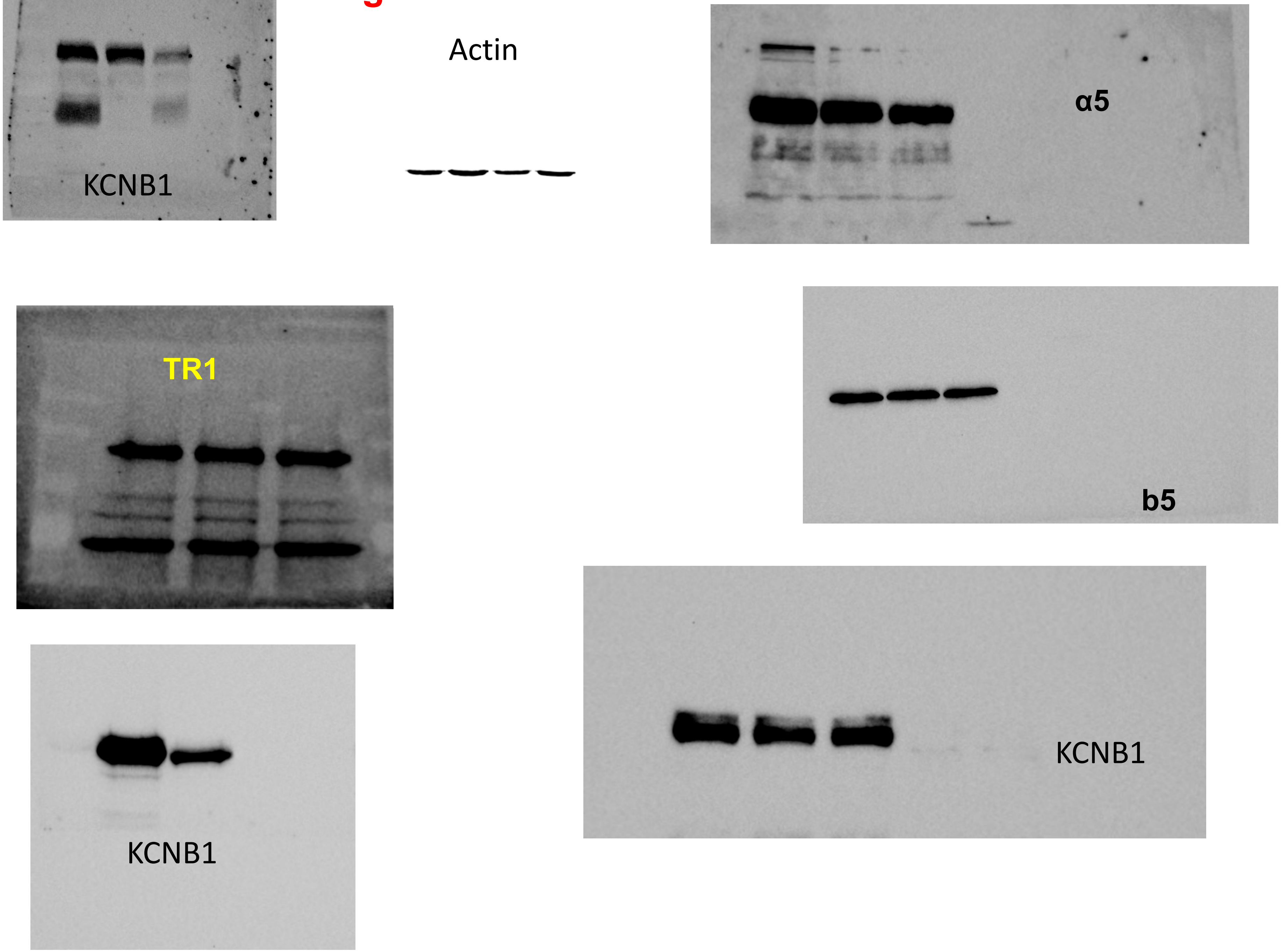

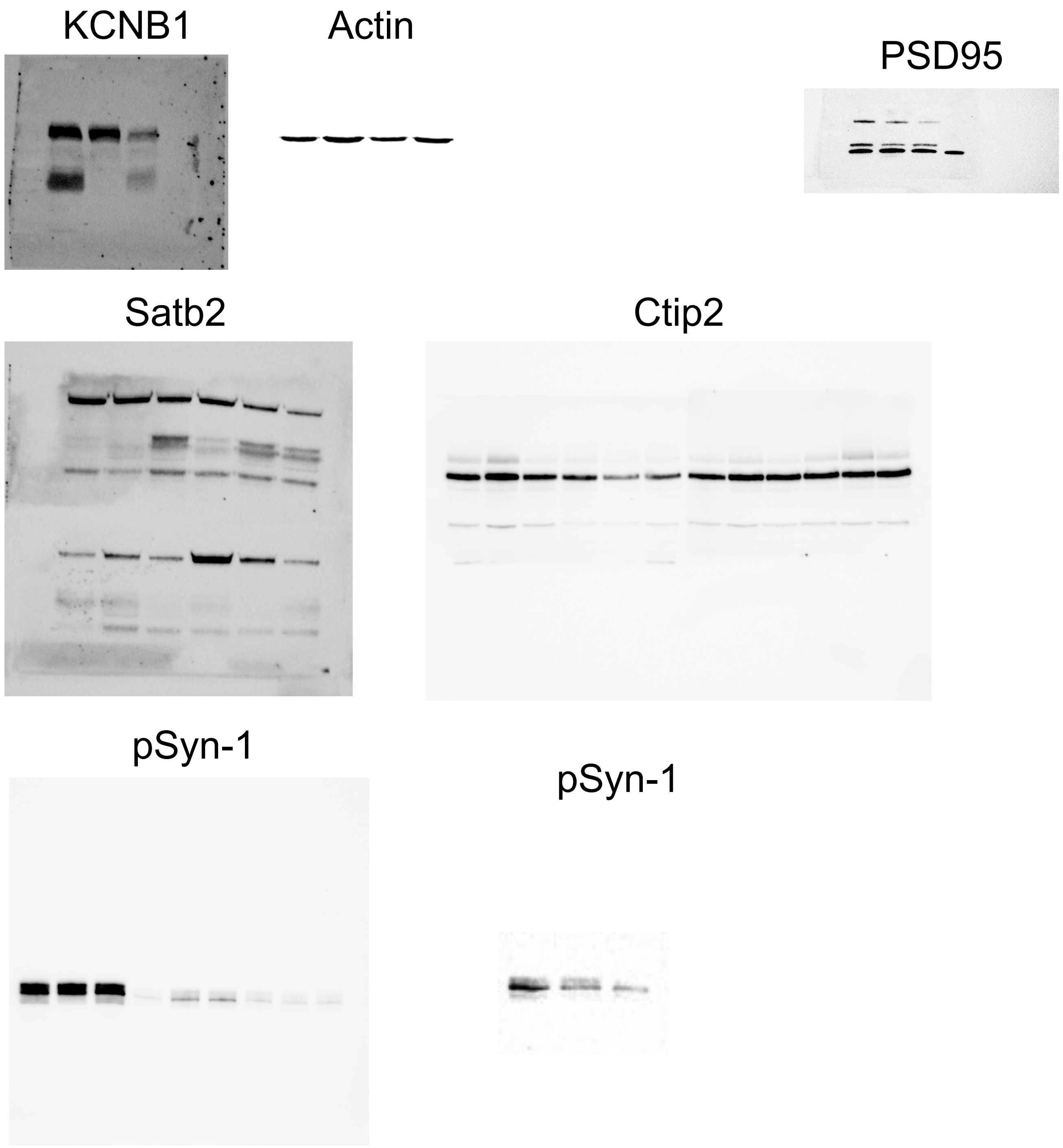
KCNB1
Actin
PSD95
Satb2
Ctip2
pSyn-1
pSyn-1

PSD95

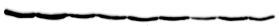

Both WTx3 Homo x3 Null x3

Syn-1

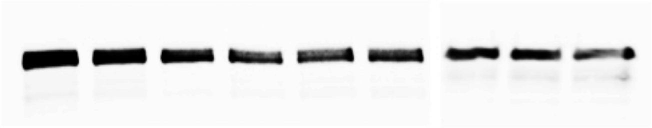

Syn-1

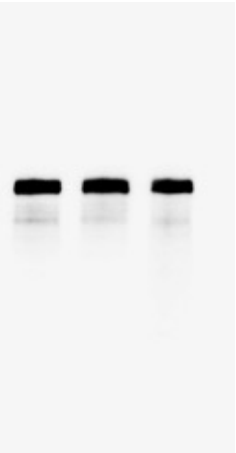

Actin

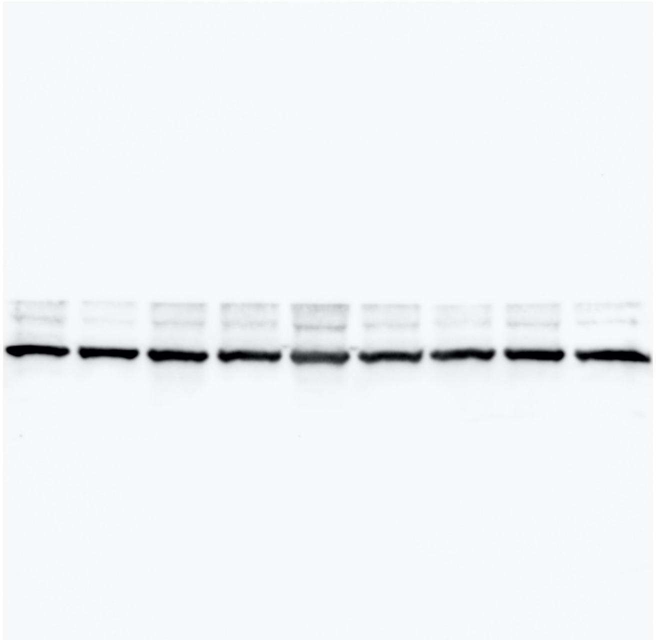

Actin

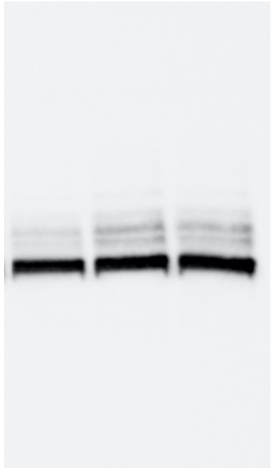

Supplement: Supplementary file 2 — uncropped Western blots [file 41418_2022_1072_MOESM2_ESM.pdf]
